# Transmission of ALS pathogenesis by the cerebrospinal fluid

**DOI:** 10.1186/s40478-020-00943-4

**Published:** 2020-05-07

**Authors:** Pooja Shree Mishra, Hejer Boutej, Geneviève Soucy, Christine Bareil, Sunny Kumar, Vincent Picher-Martel, Nicolas Dupré, Jasna Kriz, Jean-Pierre Julien

**Affiliations:** 1 CERVO Brain Research Centre, 2601 Chemin de la Canardière, Québec, Québec G1J 2G3 Canada; 2 Department of Psychiatry and Neuroscience, Faculty of Medicine, Université Laval, Québec City, G1V 0A6 Canada; 3 Division of Neurosciences, University Hospital Center of Quebec, Laval University, Quebec City, G1V 4G2 Canada

**Keywords:** TDP43 proteinopathy, Sporadic ALS, Mouse model, Translational changes, Neurofilament, Inflammation

## Abstract

To test the hypothesis that the cerebrospinal fluid (CSF) could provide a spreading route for pathogenesis of amyotrophic lateral sclerosis (ALS), we have examined the effects of intraventricular infusion during 2 weeks of pooled CSF samples from sporadic ALS patients or control CSF samples into transgenic mice expressing human TDP43^WT^ which do not develop pathological phenotypes. Infusion of ALS-CSF, but not of control CSF, triggered motor and cognitive dysfunction, as well as ALS-like pathological changes including TDP43 proteinopathy, neurofilament disorganization and neuroinflammation. In addition, the neuron-specific translational profiles from peptide analyses of immunoprecipitated ribosomes revealed dysregulation of multiple protein networks in response to ALS-CSF altering cytoskeletal organization, vesicle trafficking, mitochondrial function, and cell metabolism. With normal mice, similar ALS-CSF infusion induced mild motor dysfunction but without significant TDP43 pathology in spinal neurons. We conclude that the CSF from sporadic ALS contains factors that can transmit and disseminate disease including TDP43 proteinopathy into appropriate recipient animal model expressing human TDP43. These findings open new research avenues for the discovery of etiogenic factors for sporadic ALS and for the testing of drugs aiming to neutralize the ALS-CSF toxicity.

## Introduction

Amyotrophic Lateral Sclerosis (ALS) is characterized by relentlessly progressive degeneration of motor pathways and severe muscular atrophy; with a mean survival rate of 3–5 years following onset. The incidence of amyotrophic lateral sclerosis is 1 to 3 per 100,000 person/year with a peak age of onset being 58–63 years for the sporadic variant and 47–52 years for the familial cases [35, 48]. The disease has classically been described with the clinical representation of both upper, as well as lower motor neurons, from the motor cortex, brain stem and spinal cord. However, increasing evidence supports the involvement of non-motor components including executive, behavioral and language impairment and fronto-temporal dementia (FTD), cementing its status as a multi system neurodegenerative disorder [27, 59].

Pathological hallmarks of ALS include motor neuronal death, muscle atrophy, as well as severe, chronic neuroinflammation and glial involvement. At the cellular level, cytoskeletal derangements and axonal defects, organellar damage, glutamate excitotoxicity, oxidative stress, and alterations in RNA metabolism have been observed [27, 35, 56]. In familial cases of ALS, mutations in several genes have been implicated including *SOD1, C9ORF72, ATXN2, OPTN, VCP, VAPB, DCTN1,* Fig. 4*, UBQLN2, SQSTM1* and *TARDBP* encoding TAR DNA Binding protein 43 (TDP43) [8]. Some of the genetic aberrations implicated in familial ALS have also been found at low frequency in sporadic ALS (sALS) [8, 36, 64]. For the majority of sALS which constitutes ~ 90% of the total ALS cases, the etiologies remain unknown. Various environmental factors, including physical insults and injury, nutrition, smoking and ethnicity have been proposed but no single factor has been sufficient to explain the pathogenesis of sALS [10, 18, 33].

A hallmark of ALS is the abnormal cytoplasmic aggregation of TDP43 in degenerating neurons [40]. TDP43 is a heterogeneous nuclear ribonucleoprotein (hnRNP) involved in RNA splicing, DNA repair processes, chromatin condensation and mRNA translation [11, 39, 51]. Certain pathological variations of TDP43 overlap in ALS and FTD, unifying both the disorders under the term ‘TDP43 proteinopathy’. These include alternately spliced, truncated, ubiquitinated, SUMOylated, phosphorylated and acetylated forms of TDP43 in the spinal cord and brain, often associated with neuronal and glial mislocalization and aggregation of TDP43 species [21, 51]. Moreover, some reports provided evidence of prion-like propagation of TDP43 pathology through self-seeding and exosome transmission [32, 51]. In line with the concept of disease propagation by protein misfolding and aggregation was the report that human hTDP43^WT^ potentiated TDP43 pathology with ensuing lethal phenotype when co-expressed with ALS-linked mutant TDP43^Q331K^ in transgenic mice [46].

It has been proposed that key toxic factors in the spread of ALS disease reside in the cerebro-spinal fluid (CSF) [57]. Indeed, CSF samples from ALS patients exhibited altered proteome when compared to CSF from healthy controls with potential pathogenicity of the altered components [25, 63, 67, 68]. Acute infusion of CSF from ALS (ALS-CSF) into rats provided further evidence of CSF etiogenic factors causing neuronal alterations [26, 50], neuroinflammation [44, 45], muscular abnormalities [54] as well as electrophysiological alterations [52, 53].

To test the hypothesis that CSF constitutes a route of ALS dissemination including TDP43 pathology, we have performed chronic intracerebroventricular infusion of pooled CSF samples from sporadic ALS patients or from non-ALS control samples into transgenic mice expressing human TDP43^WT^ which do not develop pathological phenotypes [46]. Here, we report that a 14-day-infusion of ALS-CSF in hTDP43^WT^mice triggered motor and cognitive dysfunction as well as ALS-like pathological changes, including cytoplasmic TDP43 proteinopathy, neurofilament abnormalities and neuroinflammation. Moreover, neuron-specific translational profiles from the brain revealed dysregulation of multiple protein networks in response to ALS-CSF affecting cytoskeletal organization, vesicle trafficking, mitochondrial function, and cell metabolism.

## Materials and methods

### CSF sample collection and study groups

CSF samples were collected from patients diagnosed with ALS (ALS-CSF; *n* = 10, based on the revised El- Escorial criteria [7], after obtaining informed consent in accordance with the institutional human ethics committee guidelines. The samples included CSF from 7 males and 3 females with a mean age of 58.3 ± 8.85 years. CSF samples from age-matched subjects with Normal pressure Hydrocephalus pathology were also collected and studied as disease control (NALS-CSF, *n* = 5; mean age 67.6 ± 9.63 years). We also collected postmortem human brain samples from ALS patients (n = 5) and healthy controls (*n* = 4). For the present study, ALS and NALS CSF samples were separately pooled and investigated in-vivo or in-vitro.

### CSF exposure in NSC-34-hTDP43WT-HA-luc p65 cells

CSF exposure was carried out on the NSC-34 cells (NSC-34-hTDP43WT-HA-luc p65) stably transfected with hTDP43^WT^-HA, as well as with pGL4.32 [luc2p/NF-κB-RE/Hygro] (Promega Corp., Madison, USA). The cells were plated at a density of 2.5X10^4^ cells/ml and allowed to differentiate till 70–80% confluence was reached. The cultures were then exposed to different experimental conditions, namely normal controls propagated in media alone (NC), as well as cultures exposed to 10% v/v NALS-CSF and ALS-CSF respectively, as reported earlier [69]. These cultures were then used to either study the luciferase activity, or TDP43 mislocalization.

### Luciferase activity

The NSC-34-hTDP43WT-HA-luc p65 cells were plated in 24 well plates and subjected to the CSF exposure. After 48 h, cells were lysed with glo-lysis buffer (Promega Corp., Madison, USA). The lysates were centrifuged at 14,000 g and 50 μl of the supernatant was added to 96 well plates in triplicates for each sample. An equal volume of Bright Glo luciferase assay system (Promega Corp., Madison, USA) was added to each well and the luciferase activity was measured as luminescence (Enspire, Perkin Elmers Waltham, USA). The activity was normalized to the total protein for each sample and the changes were plotted as fold changes compared to the control.

### DNA constructs, generation of transgenic mice, and genotyping

The transgenic hTDP43^WT^ mice used in the study were kindly given by Mitchell and colleagues [46]. The NFL-HA-mRFP1-RPL10a transgenic mice were generated. First, HA-mRFP1 fragment was obtained by PCR using the following primers: 5′ primer: 5′-GGG ACG ACG AAT TCG GAG GCA GCA TGT ACC CAT ACG ATG TTC CAG ATT ACG CTG CCT CCT CCG AGG ACG T-3′ and 3′ primer: 5′-GGG ACG ACG GAT CCG GCG CCG GTG GAG TGG CGG CCC-3′. Then, the amplified fragment was introduced into pBluescript KS+ plasmid into corresponding restriction sites. A 2.5 Kb BamHI /NotI fragment corresponding to the genomic DNA of 60s ribosomal protein L10a (RPL10a) was introduced into corresponding restriction sites of pBSKS-HA-mRFP1 recombinant vector. A 3.4 Kb XhoI/XhoI fragment corresponding to the HA-mRFP1-mRPL10a transgene was introduced into pSKhNFL plasmid instead of the exon 1. As described [9] this plasmid contains human NFL gene sequences including − 292 bp of 5′ flanking sequences and intron sequences sufficient to drive NFL expression in the nervous tissues of adult transgenic mice. To facilitate the digestion of the transgene, a KpnI restriction site was added to the pSKhNFL plasmid at the position 6711 bp. The integrity of the final construct was verified by sequencing.

For the generation of the NFL-HA-mRFP1-RPL10a transgenic mice, a KpnI-KpnI DNA fragment of 9.0 kb was isolated on agarose gel for microinjection. The transgenic mice named NFLrRFP were viable and did not develop overt phenotypes and were genotyped by PCR amplification. For genotyping, a 179 bp fragment from the mRFP1 gene is amplified from the NFLrRFP transgenic mice and not from the wild type mice. The PCR was performed on ear punch samples using the 5′ mRFP1-GEN primer: 5′-GACCGCCAAGCTGAAGGTGA-3′ and the 3′ mRFP1-GEN primer: 5′-CCGTCCTCGAAGTTCATCAC-3′. The mice line thus obtained was crossed with the transgenic hTDP43^WT^ mice line to obtain the double transgenic mice named NFLrRFPxh-TDP43^WT^ and confirmed by genotyping as previously described. The forward and reverse primers used to confirm h-TDP43^WT^ genotype were 5′-GGATGAGCTGCAGTTCT-3′ and 5′-TGCCCATCATACCCCAACTG-3′, respectively.

### Surgical procedure and CSF administration

The mice were randomly assigned to the experimental groups, taking gender and age into consideration. For i.c.v. delivery of PBS, as well as pooled NALS and ALS CSF samples, mice (mean age 230.41 ± 37.7 days) were anesthetized with isoflurane and placed in a stereotaxic apparatus (David Kopf Instruments, Tujunga, USA). The right lateral ventricle was reached (1.50 mm lateral, − 1.00 mm antero-posterior and − 2.00 dorsoventral from Bregma) with a 30-gauge stainless steel cannula (Roanoke, VA) that was connected to an Alzet osmotic mini-pump model 1002 (Durect, Cupertino, USA). The sample administration was carried out for 2 weeks at a rate of 0.25ul/hr. On the 15th day, the mice were sacrificed to investigate the molecular and histological pathology.

### Behavioral analyses

The mice were monitored for the changes in body weight and extension reflexes before and after 14 days of CSF administration. To measure the extension reflex, the mice were suspended by the tail for 30 s and the degree of motor deficit was scored on a scale from 0 to 2. A normal extension reflex in both hind limbs was given a score of 0. The severity was increasingly ranked on a scale of 0.5 (mild) to 1 (severe) for each hindlimb, thus allowing a maximum score of 2, reflecting a complete absence of extension reflexes in both the hind limbs.

On the 15th day, open-field analysis was done to assess the locomotor activity [62]. The mice were placed individually into the open-field testing chamber in dark to minimize anxiety. The movements, horizontal and vertical activity, as well as stereotypic count were video recorded for 30 min in 6 bins of 5 min, resulting into 6 different trials for each animal. After the test, the mice were returned to their home cage. The data was generated by using the VersaMax System (AccuScan Instruments, Inc., Columbus, Ohio, USA) and was directly taken for analysis. To evaluate the gait pattern, catwalk analysis was done based on previously reported method [47]. To assess the memory impairment, the mice were subjected to novel object recognition (NOR) assay performed as a 3-day test, in a 20 × 50 × 30 cm Plexiglass box for 5 min per session [32]. Briefly, on the first day the animals were familiarized with the cage, and on the second day, two identical objects were placed equidistantly, while the mice were allowed to interact with both the objects for 5 min. On the third day, one of the objects was replaced with novel object and the percent time spent around the familiar and novel object was used to measure the preference of the mice for the novel object (a) as well as the total exploration time (b).

### Tissue collection

Following 14 days of ICV infusion, the mice were deeply anesthetized with 10 μl/g pentobarbital 12 mg/ml prior to sacrificing. The brain and spinal cord tissues were quickly dissected, snap frozen and stored at − 80 °C to be further used to validate the results by western blot analysis and biochemical assays. Fresh Tibialis anterior (TA) muscle tissues were embedded in OCT medium and snap frozen to obtain 16 μm thick cryosections for Hematoxylin and Eosin (H and E) staining. Another set of mice was transcardially perfused with PBS followed by fixation with 4% paraformaldehyde. Dissected brain, spinal cord and muscle tissues were post fixed overnight in 4% paraformaldehyde and equilibrated in a solution of PBS-sucrose (30%) for 48 h or until the tissues sank. Spinal cord and brain tissues were then cut in 25 μm thick sections with a Leica frozen microtome and stored in a cryoprotective solution at − 20 °C until further use. The muscle tissues were cut in 16 μm thick cryosections on a cryostat and stored directly at − 20 °C.

### Immunocytochemistry, immunohistochemistry and quantitative analysis

For immunocytochemical analysis, the NSC-34 cells grown in 24well plates were fixed using 4% PFA for 10 min, equilibrated with PBS and blocked with 3% normal goat serum in 0.1 M PBS (pH 7.4). The cells were then incubated with TDP43 antibody followed by anti-Rb secondary antibody conjugated with Alexa488 (Table 1), washed, and mounted for visualization. Total integrated density (ID) of TDP43 for individual neurons was measured using freehand selection feature of ImageJ to mark cell soma/ nucleus as the regions of interests (ROIs). The nuclear TDP43 ID for each neuron was determined by analyzing the intensity corresponding to the nuclear ROI based on the blue (DAPI) channel for each neuron. Cytoplasmic TDP43 ID was determined by subtracting the nuclear TDP43 ID from the ID of the entire soma and following this, the nuclear to cytoplasmic intensity ratio (N/C ratio) was plotted. At least 3 sections per mice were considered for comparison across the groups. H and E staining for muscle tissue was done in a slightly modified way from the previously described protocol [70]. Briefly, fresh, unfixed muscle sections were incubated with hematoxylin solution in a staining jar for 1 min followed by 10 min in tap water to stain the nuclei. The sections were then stained with Eosin solution for 30 s and washed thoroughly under tap water. Following this, the slides were passed in succession through 70% ethanol for 20 s(× 2), 90% ethanol for 20 s (× 2), 100% ethanol for 1 min (× 2), and xylene for 3 min. The stained slides were dried and mounted with a xylene-based mounting medium. Images were captured at 20 X with a Leica DMI 6000B microscope. The average cross-sectional area of the muscles was measured by using the particle analysis feature of ImageJ. Briefly, the images were converted to 8-bit binary with Huang thresholding, following noise removal and applying a band pass filter range of 40–3 pixels to properly define the structure. The area for each fiber/field and average size was measured and a frequency distribution curve was generated for the cross-sectional area of the muscle fibers. At least 4 sections per mice were considered for analysis.

**Table 1:** List of the antibodies used in the study

Antibody	Species	Company	#Cat.	Dilution		Temp	Hrs
				IF	WB		
Anti-Actin	Rabbit polyclonal	Sigma	A2668	–	1:1000	4 °C	24
Anti-Arginase1	Rabbit polyclonal	SCBT	SC20150	–	1:1000	4 °C	24
Anti-ChAT	Rabbit polyclonal	Millipore	AB143	–	1:1000	4 °C	24
Anti-Chit-1	Rabbit polyclonal	SCBT	SC46853	1:500	1:1000	4 °C	24
Anti-Galectin3	Rabbit polyclonal	Abcam	AB76245	1:500	1:1000	4 °C	24
Anti-InA	Rabbit polyclonal	Millipore	AB5354	1:500	1:1000	4 °C	24
Anti-Mitofilin	Rabbit polyclonal	Invitrogen	PA3870	1:500	–	4 °C	24
Anti-NfL	Rabbit polyclonal	Millipore	AB5294	1:500	1:1000	4 °C	24
Anti-NfM	Rabbit polyclonal	Millipore	AB1981	–	1:1000	4 °C	24
Anti-TDP43	Rabbit polyclonal	Proteintech	#10782	1:800	1:5000	4 °C	24
Anti-TDP43 (pSer 409/410)	Rabbit polyclonal	Cosmobio	TIP-PTD-P01	–	1:1000	4 °C	24
Anti-phospho-p65	Rabbit monoclonal	Cell signalling	#4764S	1:500	–	4 °C	24
Anti-Tuj1	Rabbit monoclonal	Covance	MRB435P	1:800	–	4 °C	24
Anti-ATP5A	Mouse monoclonal	SCBT	SC-136178	–	1:1000	4 °C	24
Anti-GAPDH	Mouse monoclonal	SCBT	SC32233	–	1:2000	4 °C	24
Anti-GFAP	Mouse monoclonal	Cell signalling	#3670	1:800	1:2000	4 °C	24
Anti-HSPA9	Mouse monoclonal	SCBT	SC-133137	–	1:1000	4 °C	24
Anti-human TDP43	Mouse monoclonal	Abnova	H000023435	1:500	1:1000	4 °C	24
Anti-NeuN	Mouse monoclonal	Millipore	AB377	1:500	1:1000	4 °C	24
Anti-Peripherin	Mouse monoclonal	Millipore	AB1527	1:500	1:1000	4 °C	24
Anti-PGC1α	Mouse monoclonal	SCBT	SC-517380	1:500	–	4 °C	24
Anti-Mouse IgG- HRP	Goat polyclonal	Invitrogen	G-21040	–	1:5000	RT	1
Anti-mouse IgG- IRdye-680	Goat polyclonal	Li-Cor	#926–8070	–	1:10,000	RT	1
Anti-rabbit IgG- Alexa 488	Goat polyclonal	Invitrogen	# A32731	1:500	–	RT	1
Anti-rabbit IgG- IRdye-800	Goat polyclonal	Li-Cor	#926–32,211	–	1:10,000	RT	1
Anti-rabbit IgG-Alexa 568	Goat polyclonal	Invitrogen	# A-11036	1:500	–	RT	1
Bungarotoxin-Alexa594		Invitrogen	B13423	1:500	–	4 °C	24

Nissl staining was done according to a published protocol [49]. Briefly, slides containing fixed spinal cord sections were differentially dehydrated in alcohol series followed by rehydration. Following this, the sections were incubated in 1% cresyl violet stain for 1 min, followed by differentiation with 100% ethanol in glacial acetic acid for 5 s. The sections were then rehydrated and mounted using DPX mounting medium (Sigma-Aldrich, Inc., St. Louis, USA). The quantification was carried out using the particle analysis feature of ImageJ as previously described in this section, using a size-based filter above 250 μm^2^ to select motor neurons for the analysis [49].

For the immunofluorescence studies, the spinal cord and brain sections were subjected to antigen retrieval in 0.01 M citrate buffer (pH 6.0), washed twice, equilibrated with PBS, and blocked with 3% normal goat serum in 0.2 M PBS (pH 7.4). The sections were then incubated with the primary antibodies of interest (Table 1) overnight in PBS-Triton-X, followed by appropriate fluorophore tagged secondary antibodies for 2 h at RT. The sections were thoroughly washed with PBST and incubated with DAPI for 5 min. Following this, the sections were mounted with the help of anti-fade mounting media and visualized under a confocal laser scanning microscope (Nikon, Tokyo, Japan). The mitofilin staining was quantified using the particle analysis feature for calculating average size of particles per field for each group. At least 3 sections were considered per mice for the analysis.

The muscles were similarly immunostained for the neuromuscular junctions (NMJ) with α-Bungarotoxin antibody conjugated with alexa-594, as well as Neurofilament (NF) antibody followed by Alexa 488 conjugated secondary antibody. The sections were then mounted and proceeded for visualization. Analysis was carried out by counting the number of innervated, as well as fully or partially denervated NMJs from at least 4 sections per mice, and at least 3 mice per group.

### Immunoblotting

The snap frozen brain (cortex and hippocampus) and spinal cord tissues were processed to obtain protein lysates for various immunoblotting experiments. The tissues were suspended in RIPA buffer (50 mM Tris, pH 7.4, 1 mM EDTA, 150 mM NaCl, 1% NP-40 supplemented with 0.5% Sodium deoxycholate, 0.1% SDS, as well as protease and phosphatase inhibitors) and sonicated. Insoluble and soluble fractions were obtained from the brain and spinal cord tissues as previously described [13]. The proteins were quantified using the bicinchoninic acid (BCA) method (Bio-Rad Laboratories, Hercules, USA). The protein extracts were separated by SDS-PAGE and transferred onto a low fluorescence nitrocellulose membrane (LI-COR Biosciences, Lincoln, USA), and probed with antibodies against the proteins of interest (Table 1). The blots were then incubated with appropriate IR-conjugated secondary antibodies (LI-COR Biosciences, Lincoln, USA) and visualized using the Odyssey imaging software (LI-COR Biosciences, USA). For analysis, the band intensities were quantified using the Image Studio Lite 5.0 software (LI-COR Biosciences, Lincoln, USA). For the total protein quantitation, the blots were stained for total protein using either the stain free gels (Bio-Rad Laboratories, Hercules, USA), Revert total protein stain (LI-COR Biosciences, Lincoln, USA), or Ponceau S. Individual lanes were quantified using the lane profile feature of the Image lab software (Bio-Rad Laboratories, Hercules, USA. The values were normalized either to loading control (GAPDH or Actin) or to the total protein present in the individual lanes.

### Biochemical assays

To obtain lysates for the Triose Phosphate Isomerase (TPI) Activity Assay, 10 mg of tissue was carefully homogenized in 200ul of ice cold TPI Assay Buffer with a Dounce homogenizer and kept on ice for 10 min. The samples were then centrifuged for 5 min at 4 °C at 10,000 x g using a cold microcentrifuge to remove any insoluble material, and the supernatant was taken for TPI activity and total protein analysis. The TPI assay was performed using a kit (Abcam, Cambridge, USA; cat no: ab197001) according to the manufacturer’s instructions. The activity was normalized to the total protein analyzed using the BCA quantification method.

To assess the p65 activity in-vitro, Luciferase Assay was performed using the Bright-Glo Luciferase assay system (Promega Corp., Madison, USA), as previously described [49]. The relative luciferase activity (RLU) was normalized to the total protein analyzed using the BCA quantification method.

### EDTA-translational affinity purification (TRAP) protocol

To assess the neuronal molecular signatures in-vivo, we took advantage of the EDTA-TRAP approach in the hNfL-RFP mouse model and extracted the freshly translated peptides following 14- day administration of PBS or pooled CSF across various groups. For each of the biological triplicates, we pooled the hippocampi and cortices of two mice (*n* = 6) to achieve an optimal concentration for the experiment. For PBS, technical triplicates were taken. Cortical and hippocampal tissues were collected in ice cold dissection buffer and quickly homogenized in (10% w/v) in tissue lysis buffer. The samples were centrifuged at 2000 g for 10 min at 4 °C and to the supernatant, 10% NP-40 and 300 mM DHPC (1/9 sample volume each) were added. Following this, the samples were incubated for 30 min at 4 °C on an orbital shaker. The soluble fraction was collected by centrifugation at 20,000 g for 10 min at 4 °C and incubated with the anti-RFP agarose affinity resin (ChromoTek Planegg-Martinsried, Germany) and incubated overnight at 4 °C on an orbital shaker. Following this, the beads were recovered by centrifugation at 4oC for 5 min 3000 rpm and washed (3x) with high-salt buffer (20 mM HEPES-KOH [pH 7.3], 350 mM KCl, 12 mM MgCl2, 1% NP-40, 0.5 mM DTT, and 100 mg/mL cycloheximide). The beads were then resuspended for 30 min at RT in EDTA-elution buffer (10 mM HEPES-KOH [pH 7.3], 150 mM KCl, 5 mM MgCl2, 20 mM EDTA, and protease inhibitors) to release the nascent peptides. The supernatant was recovered by centrifugation at 5000 rpm for 15 min and contained the released peptides. The peptides were quantified using the Bradford assay and an equal amount of proteins was then analyzed by mass spectrometry using Orbitrap fusion mass spectrometer (Thermo Fisher Scientific, San Jose, USA).

### Mass spectrometry analysis

The analysis was carried out as previously described [6]. Briefly, the samples were concentrated on desalting column Amicon 3 kDa (Millipore, Burlington, USA), and given 3 washes with ammonium bicarbonate 50 mM. Equal amounts of protein were solubilized in the denaturation buffer, followed by heating to 95 °C for 5 min in a solution of DTT and iodoacetamide, and addition of 1 μg with overnight incubation at 37 °C. Subsequent steps of 10 min RT incubation and 5 min RT centrifugation at16000g were followed to eliminate the precipitated sodium deoxycholate. The supernatant was desalted on C18 Empore filter (Sigma-Aldrich, Inc., St. Louis, USA). Peptides were eluted in 80% Acetonitrile (ACN) with 0.1% (v:v) trifluoroacetic acid (TFA), and dried in speed vac.

750 ng of peptide sample triplicates from each group were injected and separated by online reversed-phase (RP) nanoscale capillary liquid chromatography (nanoLC) and processed for analysis by electrospray mass spectrometry (ESI MS/MS). The technique was carried out using a Dionex UltiMate 3000 nanoRSLC chromatography system (Thermo Fisher Scientific / Dionex Softron GmbH, Germering, Germany) connected to an Orbitrap Fusion mass spectrometer (Thermo Fisher Scientific, San Jose, USA) with a nanoelectrospray ion source. With Thermo XCalibur software version 3.0.63. Full scan mass spectra (350 to 1800 m/z) were acquired using an AGC target of 4e5, a maximum injection time of 50 ms and a resolution of 120,000. Each MS scan was followed by acquisition of fragmentation MSMS spectra of the most intense ions for a total cycle time of 3 s (top speed mode). Mass spectrometry analyses were performed by the Proteomics platform of the Eastern Quebec Genomic Center, CHU de Quebec, Canada. Database searching and Label Free Quantification Spectra were searched against a mouse proteins database (UniprotKB – taxonomy *Mus musculus* – 84,675 sequences) using the Andromeda module of MaxQuant software v. 1.5.0.25 [15]. Only the fold change higher or lower than 1.2 with *p* < 0.05 were considered significantly dysregulated in the present study.

### Translatome analysis

Differentially expressed peptides (genes) were used to generate biological networks using Gene Ontology (GO) biological and molecular process, using the ClueGo application (version2.5.6) within the Cytoscape environment (3.7.2) The network analysis for the data was carried out using the ClueGo application of the Cytoscape software (version 3.71) [5]. The GO interval was selected for a range of 4 to 11 with the two-sided (Enrichment/Depletion) tests, Benjamini-Hochberg statistics corrected for mid-*P* values, and Kappa score at 0.5. Only the interactions having a *p*-value < 0.05 were considered for the analysis. The leading group term was based on % genes/term vs cluster.

### Study approval

All the experimental procedures were approved by the Laval University animal care ethics committee (2016060–2) and are in accordance with the Guide to the Care and Use of Experimental Animals of the Canadian Council on Animal Care. The CSF samples were collected after obtaining informed, written consent in accordance with the institutional human ethics committee guidelines.

### Statistics

The experiments were conducted with biological replicates and were also technically replicated except in behavioral assays. Statistical analyses were performed with GraphPad Prism 8.0 (GraphPad, Inc., San Diego, USA), using a one or two-way ANOVA with Tukey’s post-hoc test, or Student’s t-test, unless otherwise specified. The results were expressed as mean ± SEM. The results were considered significant when * *p* < 0.05, **p < 0.01, ***p < 0.001, ****p < 0.0001.

## Results

### Exposure to ALS-CSF induced NF-κB activation and TDP43 mislocalization in-vitro and in-vivo

We studied the effect of ALS-CSF samples pooled from 7 males and 3 female ALS subjects (mean age 58.3 ± 8.85 years) after obtaining informed consent. The mean duration was 2.25 ± 1.47 years and the pathology ranged between mild to severe (ALSFRS-R scores, 38.7 ± 5.96), with 1 of the 10 subjects exhibiting fast disease progression. These samples were compared against 5 age-matched NALS-CSF samples (mean age 67.6 ± 9.63 years) collected from the patients with normal pressure hydrocephalus pathology.

**Fig. 1:**
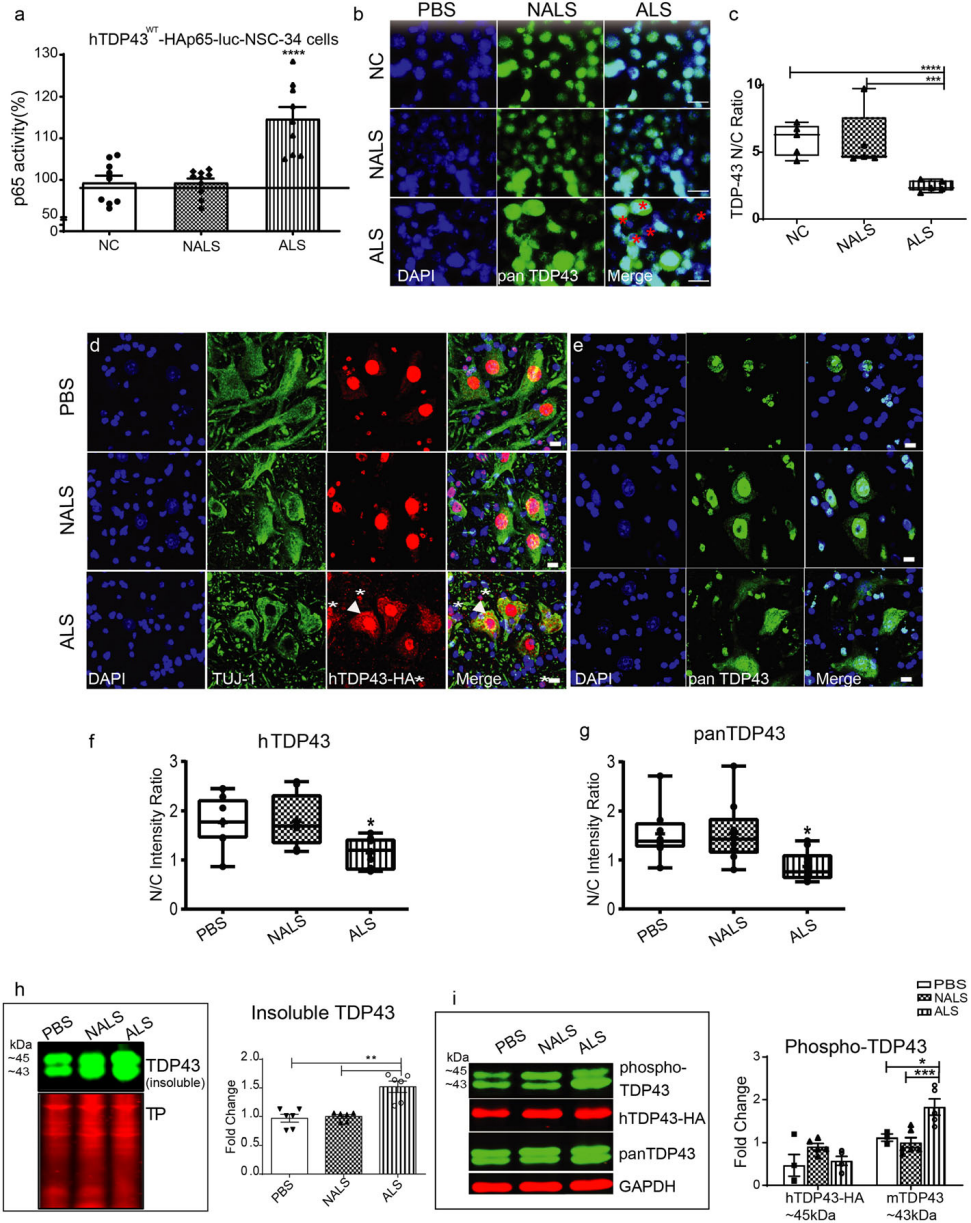
ALS-CSF induced NF-κB activation and TDP43 mislocalization. (a) Increased NF-κB activity in NSC-34 cells exposed to ALS-CSF (*n* = 3 in triplicates). (b, c). TDP43 analysis in-vitro. Representative image panels (b) and graph (c) showing cellular location of TDP43 in the NSC-34 cells across the study groups. Note the increased cytoplasmic TDP43 (red asterisks) in the ALS panel (n = 3, each in triplicates). Scale bar = 20 μm. (d- k) TDP43 immunodetection in-vivo in hTDP43 transgenic mice infused i.c.v. with PBS, CSF from non-ALS (NALS) or ALS. Representative image panels and graph for immunodetection of human TDP43 (d, f) and of pan-TDP43 recognizing both human and mouse species (e, g). Note neuronal (d, arrowhead) and glial (d, asterisk) TDP43 cytoplasmic mislocalization in the ALS-CSF panel. (h) represents qualitative and quantitative observations for the immunoblots of TDP43 obtained from the insoluble fraction of lysates using a pan-TDP43. Since the TDP43^WT^ construct in the transgenic mice was tagged with HA, the bands for human TDP43 appeared ~ 2 kDa on top of the endogenous mouse TDP43 (~ 43 kDa) on the immunoblot (i) represents qualitative and quantitative observations, of the immunoblots stained for the phosphorylated and non-phosphorylated forms of human (~ 45 kDa) and mouse (~ 43 kDa) TDP43. (n = 3, in triplicates) Scale bar = 10 μm. Data are mean ± SEM. (*p ≤ 0.05, **p ≤ 0.01, ***p ≤ 0.001 and **** p ≤ 0.001) and fold changes are calculated compared to NALS

We studied the effects of pooled CSF samples on NF-κB activity and on cytoplasmic mislocalization of TDP43 using NSC-34 cells stably transfected with vectors expressing human TDP43^WT^ and NF-κB-p65-luciferase reporter gene (NSC-34-hTDP43^WT^-HA-luc p65) capable of expressing luciferase upon NF-κB-p65 activation [49]. NSC-34 cells were subjected to luciferase reporter assay after exposure to CSF for 48 h and luminescence measured was taken as a direct measure of NF-κB-p65 activation. The activation was found to be significantly higher (> 2-fold change) in cells exposed to ALS-CSF than to cells exposed to NALS CSF (Fig. 1a). Moreover, immunofluorescence analysis of the cellular localization of TDP43 in the NSC-34-hTDP43^WT^-HA cells exposed to ALS-CSF for 48 h demonstrated a significant reduction in the nuclear to cytoplasmic ratio of TDP43 immunofluorescence (Fig. 1b, c). These in-vitro results suggested that the CSF from ALS patients, but not from control individuals, contains factors that can trigger the NF-κB signaling pathway and the mislocalization of TDP43.

To further investigate the in-vivo effects of CSF from ALS and controls, the CSF samples were administered intracerebroventricularly (i.c.v.) with mini-osmotic pumps (Alzet pump, model 1002), at a rate of 6 μL per day for a period of 14 days in transgenic mice with neuronal expression of hTDP43^WT^. We selected this mouse model as the recipient because of potential species to species restriction in prion-like propagation of disease, as well as interspecies differences in TDP43 proteinopathy [4]. Moreover, while the co-expression of hTDP43^WT^ with a pathogenic but non-lethal TDP43^Q331K^ mutation potentiated a lethal pathology within 8 weeks, the hTDP43^WT^ mice alone did not exhibit any behavioral or molecular pathology [46]. Remarkably, immunofluorescence microscopy revealed the presence of cytoplasmic TDP43 aggregates in spinal motor neurons of hTDP43 mice administered i.c.v. with ALS-CSF, but not with control non-ALS CSF or with PBS (Fig. 1d-e). A quantitative analysis of the neuronal nuclear to cytoplasmic fluorescence intensity ratio (N/C ratio) further confirmed the abnormal mislocalization of hTDP43 (anti-human TDP43 from Abnova #H000023435) as well as panTDP43 (detecting human and mouse TDP43 from ProteinTech #10782) in spinal neurons of hTDP43 mice administered with ALS-CSF when compared to the control hTDP43 mice (Fig. 1 f, g). We have prepared insoluble fraction from the spinal cord tissue lysates and analyzed by immunoblotting the levels of aggregated TDP43. (Fig. 1h). Since the TDP43^WT^ construct in the transgenic mice was tagged with HA, the bands for human TDP43 appeared ~ 2 kDa on top of the endogenous mouse TDP43 (~ 43 kDa) on the immunoblot (h). We use a polyclonal antibody Pan-TDP43 (ProteinTech 10,782) for this immunoblot. The fold changes in Fig. 1h were calculated with the intensity of the two bands (human + mouse TDP43) detected with pan-TDP43 antibody and normalized with the total protein for each biological replicate within a group, in duplicate experiments providing 6 datapoints for each group.

The levels of the insoluble TDP43 were significantly increased (~ 50% increase) in the spinal cord from ALS-CSF administered mice when compared to controls (Fig. 1 h). The level of the phosphorylated form of TDP43 (Cosmobio, #TIP-PTD-P01), a pathological feature of ALS [17], was also significantly increased (~ 2 fold change) in hTDP43 mice infused with ALS-CSF as compared to control CSF (Fig. 1 i).

It should be noted that i.cv. infusion of ALS-CSF for 14 days (6 μl CSF/day) in non-transgenic (NTG) mice (C57BL6) did not trigger TDP43 mislocalization and aggregation in spinal neurons as was observed with hTDP43 transgenic mice (Supplementary Fig. S1). So, with this experimental paradigm, neuronal expression of human TDP43 was a requirement for development cytoplasmic TDP-43 proteinopathy. It should be noted that we did observe motor phenotypes in response to ALS-CSF with normal mice albeit the magnitude was less than with hTDP43 transgenic mice. For instance, in response to ALS-CSF, the normal mice exhibited a ~ 25% reduction in the distance traveled during the open field analysis as compared to controls (NALS-CSF), unlike the hTDP43 mice which exhibited a 50% reduction (Fig. S2 a, b). Similarly the performance decline compared to controls in the normal mice was 20% for the horizontal activity (Fig. S2 c) and non-significant for vertical activity (Fig. S2 d), whereas the hTDP43 mice exhibited ~ 35% reduced performance for the horizontal and the vertical activities (Fig. S2 c,d) Moreover, the average reduction in the cross section of muscle fibers (tibialis anterior muscle) in response to ALS-CSF was 20% for normal mice and of 33%for hTDP43 transgenic mice (Fig. S2 f, h). The ALS-CSF infusion caused full denervation of 23% of neuromuscular junctions (NMJs) in normal mice in contrast to complete denervation of 50% NMJs in the hTDP43 transgenic mice (Fig. S2 g,j).

### Weight loss, abnormal extension reflexes and motor dysfunction following i.c.v. infusion of ALS-CSF in hTDP43^WT^ transgenic mice

**Fig. 2:**
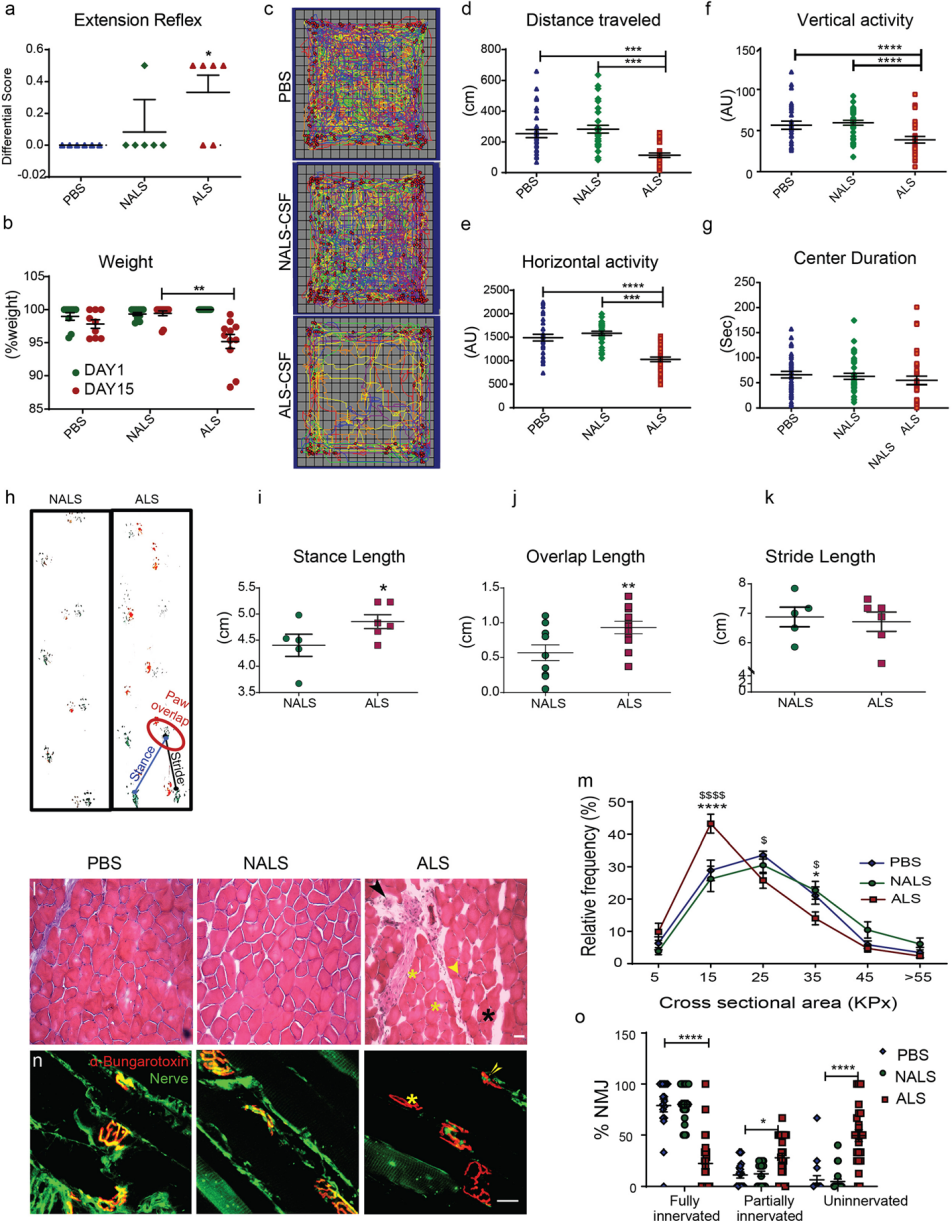
Motor dysfunction and muscular pathology in response to ALS-CSF. (a) Changes in the paw extension reflex scores from day 1 to day 15 after CSF administration (*n* = 6). (b) Changes in body weight following CSF administration (*n* = 12). (c-g) Open field test (OFT) analysis. Representative images for the locomotor pattern (c) and quantitative data for total distance traveled (d), horizontal (e) and vertical (f) activity as well as the time spent at the center (g) by the mice during OFT (n = 6). (h-k) Gait analysis. Representative images for the gait pattern (h) and quantitative data summarizing the stance length (i) paw overlap (j) and stride length (k) (n = 6). (l-o) Muscle pathology. Panel (l) and graph (m) describe the morphological changes in the H&E stained muscle tissues. Panel (n) and graph (o) highlight the complete (yellow asterisk) or partial denervation of NMJ (yellow arrowhead) (n = 3 for each different experiment). Data are mean ± SEM. (*p ≤ 0.05, **p ≤ 0.01, ***p ≤ 0.001 and **** p ≤ 0.001) and fold changes are calculated compared to NALS. Scale bar = 20 μm

We monitored the behavioral phenotypes of hTDP43^WT^ mice that were infused i.c.v. during 14 days with ALS-CSF, control NALS-CSF or PBS administered. Administration of ALS-CSF resulted in the loss of extension reflexes when compared to mice administered with control NALS-CSF or PBS (Fig. 2a). Concomitantly, these mice demonstrated a marginal (~ 5%; mean diff.: 4.72 ± 1.23%; 1.7 ± 1.45 g) but significant percent reduction in the weight of the mice from day 1 to day 15, as compared to the PBS (mean diff., 2.97 ± 1.24%; 0.033 ± 1.08 g) and NALS group (mean diff., 1.26 ± 1.29%; 0.025 ± 0.69 g) (Fig. 2b).

Open field test (OFT) was carried out [62] to assess the locomotor function of hTDP43^WT^ mice subjected to 14 days of i.c.v. infusion with CSF samples or PBS [62]. The OFT (Fig. 2c) depicted a decline greater than two foldchange in the distance traveled within 30 min (Fig. 2d) as well as significant decline in horizontal (Fig. 2e) and vertical activities (Fig. 2f) in the ALS-CSF administered hTDP43^WT^ mice as compared to the control NALS-CSF or PBS-administered mice. While the overall locomotor activity of the ALS-CSF-administered mice was significantly reduced, no difference was observed with respect to the time spent in the center region (Fig. 2g). This may suggest that the locomotor impairment of the ALS-CSF-administered mice is unlikely due to anxiety. The ambulatory dysfunction was further validated by footprint analysis (Fig. 2h) which revealed gait abnormalities, including widened stance length (Fig. 2i) and decreased paw overlap patterns in the mice administered with ALS-CSF Fig. 2j). However, there was no significant difference in the stride length between ALS-CSF- and NALS-CSF-administered hTDP43^WT^ mice (Fig. 2k).

The motor impairment of hTDP43^WT^ mice infused with ALS-CSF was further reflected by the histological alterations of muscle tissue. H and E staining of anterior tibialis (TA) muscle revealed structural changes in the form of muscle necrosis, lymphocyte infiltration (Fig. 2l, black arrowhead), adipocyte accumulation (black asterisk), multinucleation (yellow asterisk), and pyknotic nuclear clamps (yellow arrowhead) in the ALS-CSF-administered group when compared to controls, NALS-CSF- or PBS-administered groups. Moreover, the cross-sectional area of the muscle fibers was significantly reduced in the ALS-CSF administered mice (Fig. 2m). These results indicated a mild to moderate muscle pathology. We further performed an analysis of NMJs immunostained for the presynaptic (nerve, green) and postsynaptic (muscle receptors; Btx, red) components (Fig. 2 n). The analysis revealed a loss of about 60% of fully innervated NMJs in the ALS-CSF-administered mice as compared to PBS- and NALS-CSF-administered mice (Fig. 2o).

### Motor neuron death and neurofilament disorganization triggered by ALS-CSF infusion

**Fig. 3:**
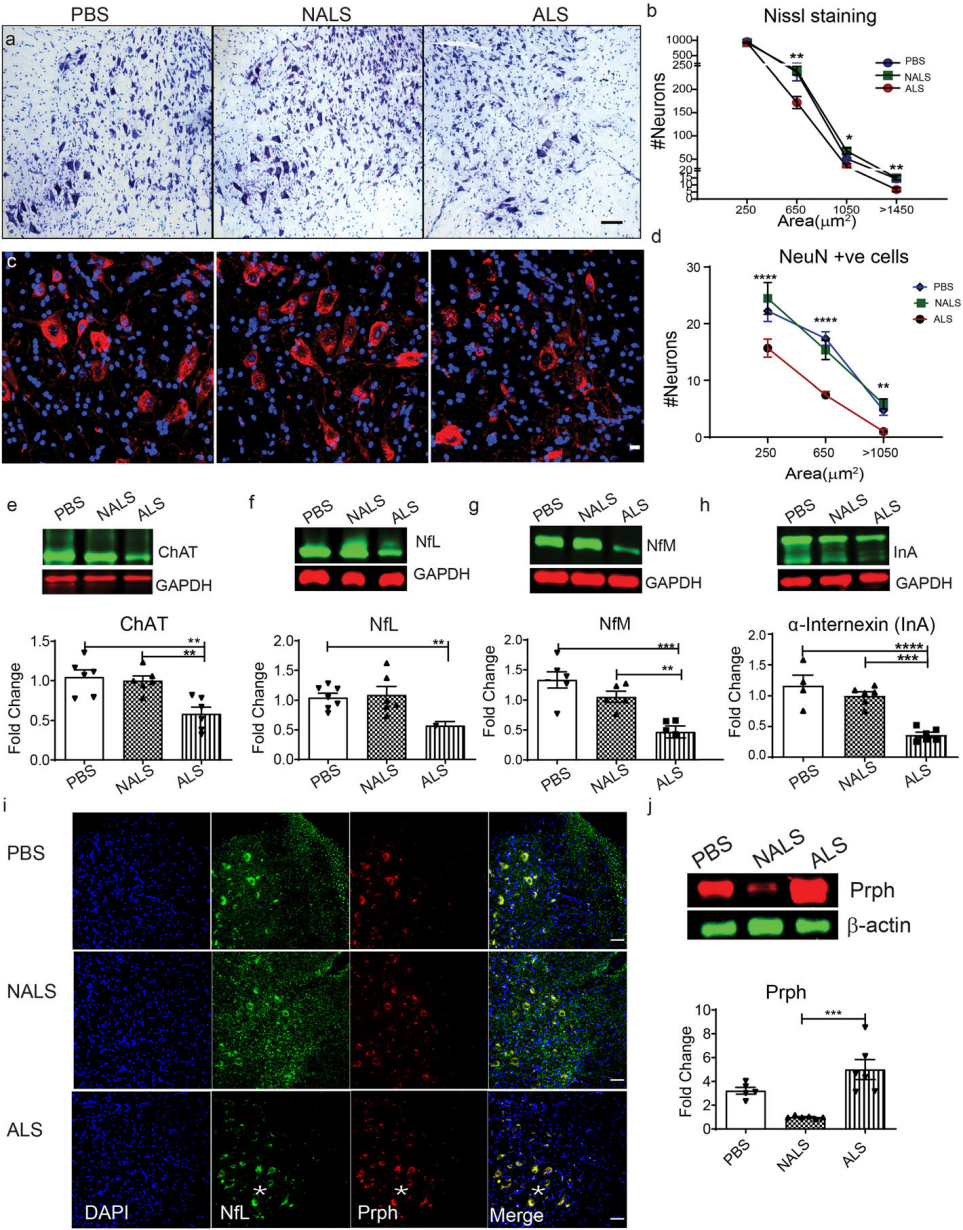
ALS-CSF induced neuronal death and neurofilament abnormalities. (a-d) Spinal cord sections stained and quantified with Cresyl violet (a, b, *n* = 3.) and NeuN (red) (c, d, n = 3), respectively. Scale bar = 50 μm (a) and 20 μm (c). (e-h) Expression patterns and quantification of the immunoblots for ChAT protein (e) as well as the neurofilaments NfL (f) NfM (g) and InA (h) in the spinal cord lysates (n = 3). (i) Spinal cord sections immunostained for NfL (green) peripherin (Prph, red) and DAPI (Blue). Note the depletion of NfL immunostaining in neurites and myelinated axons, and the co-accumulations of NfL and Prph (white asterisk, i) in neuronal cell bodies in ALS group. (j) represents the expression patterns and quantification for Prph in the immunoblots for spinal cord lysates. (n = 3), Scale bar = 20 μm. Data are mean ± SEM. (*p ≤ 0.05, **p ≤ 0.01, ***p ≤ 0.001 and **** *p* ≤ 0.0001) and fold changes are calculated when compared to NALS

We assessed the number of neurons in the ventral horn of the spinal cord samples from the different mouse groups. The cresyl violet staining showed significantly reduced number of cell bodies (~ 10%) in the area corresponding to motor neurons (> 250 μm^2^) in the ALS-CSF-administered group as compared to the controls (Fig. 3a, b). The analysis of the total number of NeuN positive cells further corroborated these results. There was a decline of ~ 35% in the NeuN positive population, which may reflect neuronal stress together with neuronal cell death (Fig. 3c, d). ChAT, the neurotransmitter involved in the neuromuscular signaling and motor neuronal marker, was also significantly downregulated by about 50% in the spinal cord from the ALS-CSF-administered mice (Fig. 3e).

Remarkably, i.c.v. infusion of ALS-CSF induced severe alterations in levels of intermediate filament (IF) proteins in the spinal cord. SDS-PAGE followed by immunoblotting revealed a 2 to 4-fold reduction in levels of neurofilament-L (NfL) (Fig. 3f), neurofilament-M (NfM) (Fig. 3g), and α-internexin (InA) (Fig. 3h) proteins in the spinal cord from ALS-CSF-administered hTDP43^WT^ mice when compared to NALS-CSF- or PBS-administered hTDP43^WT^mice. Immunofluorescence microscopy of the spinal cord from ALS-CSF treated hTDP-43^WT^ mice revealed a massive depletion of NfL immunostaining in neurites and myelinated axons (Fig. 3i, green). Interestingly, the NfL protein was co-localized in neuronal cell bodies with accumulations of peripherin (Prph) (Fig. 3i; white asterisks), a type III IF, which is known to accumulate and to form filamentous spheroids in ALS [12]. In contrast to NfL and NfM, ALS-CSF administration caused an overexpression of Prph (Fig. 3i, j). Previous transgenic mouse studies have revealed that such upregulation of peripherin in context of NfL reduction can trigger motor neuron degeneration with ALS-like phenotypes and axonal transport defects [2, 42].

### Neuroinflammation in the spinal cord of hTDP43^WT^ mice administered with ALS-CSF

**Fig. 4:**
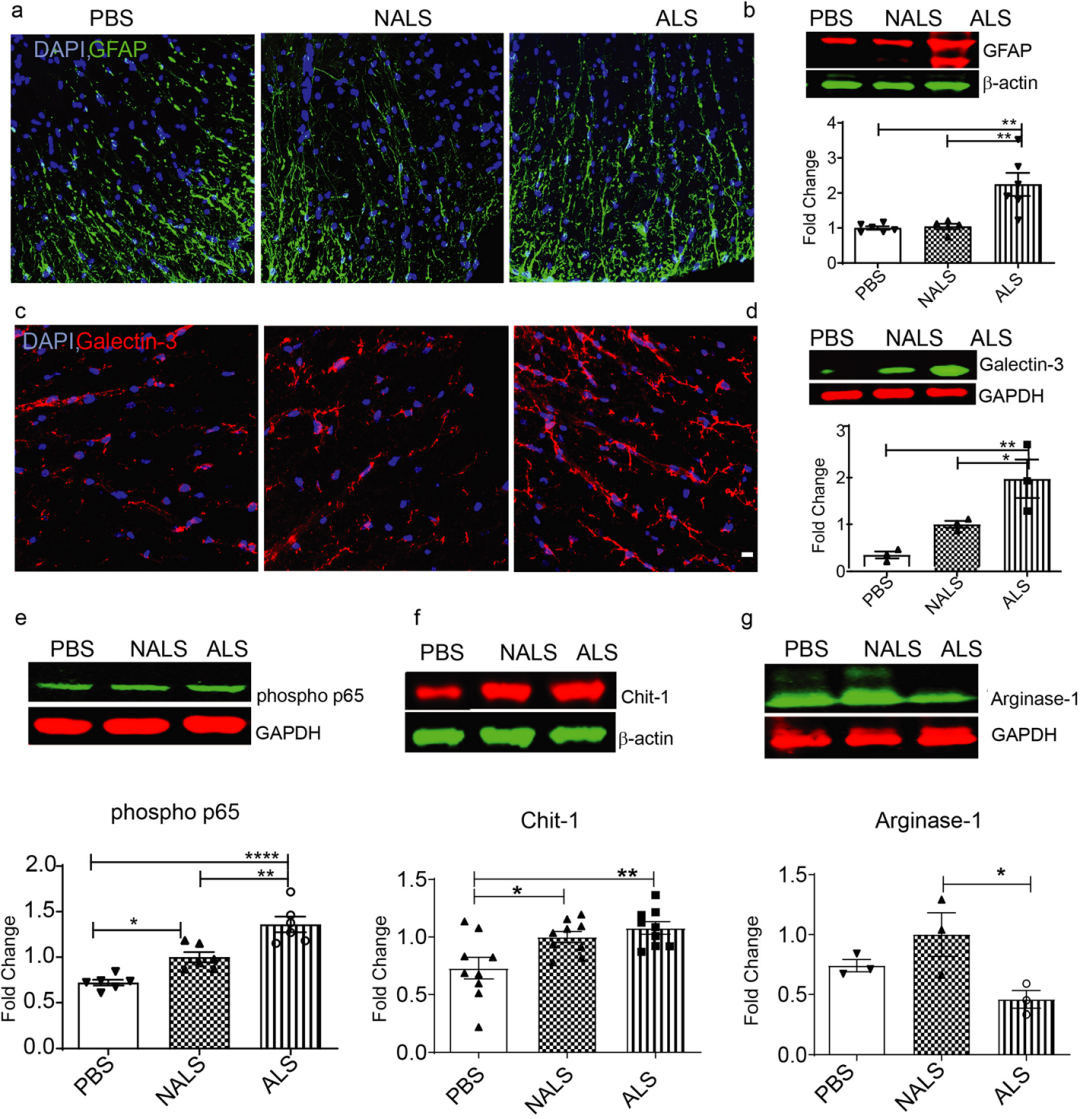
Infusion of ALS-CSF induced neuroinflammation. (a-d) Glial activation. Representative confocal images of the spinal cord sections stained with GFAP (a) and Galectin-3 (c). Expression patterns and quantification of the immunoblots for GFAP protein (b) as well as Galectin-3 (d) protein in the spinal cord lysates. (e-g) Expression patterns and quantification of the immunoblots for immune markers: phosphorylated p65 subunit of NF-κB (e), Chit-1 (f) and Arginase-1 (g) in the spinal cord lysates. Data are mean ± SEM. (*p ≤ 0.05, **p ≤ 0.01, and **** p ≤ 0.0001) and fold changes are calculated when compared to NALS. (n = 3) Scale bar = 20 μm

The hTDP43^WT^ mice administered with ALS-CSF exhibited astrogliosis and microgliosis. A qualitative assessment of the spinal cord white matter immunofluorescent stained for astrocyte and microglial markers, namely GFAP and galectin-3, revealed increased immunoreactivity towards both proteins in samples from ALS-CSF-administered mice (Fig. 4 a and c respectively). Moreover, quantitative assessment of immunoblots after SDS-PAGE of spinal cord extracts revealed a ~ 2.5-fold increase of both, GFAP (Fig. 4 b) and galectin-3 (Fig. 4 d), corroborating our qualitative microscopy observations [31]. Immunoblotting of spinal cord extracts was carried out to assess the expression pattern of various inflammatory factors commonly associated with ALS. The active phosphorylated form of NFκB-p65 was upregulated in the spinal cord of ALS-CSF-administered mice when compared to the PBS-administered or to the NALS-CSF-administered groups (Fig. 4 e). Chitotriosidase (Chit-1), a macrophage marker of inflammation, was also found to be significantly upregulated in the samples from ALS-CSF-administered mice when compared to PBS-administered controls (Fig. 4 f). However, we also observed such an increased expression of Chit-1 in the samples from NALS-CSF-administered mice (Fig. 4 f). Moreover, arginase-1, involved in the alternate activation pathway [24] was found to be predominantly downregulated exclusively in the ALS-CSF-administered group (Fig. 4 g), flagging the toxic nature of the inflammatory response associated with exposure to ALS-CSF.

### ALS-CSF triggered cognitive impairments and brain pathology in hTDP43^WT^ transgenic mice

**Fig. 5:**
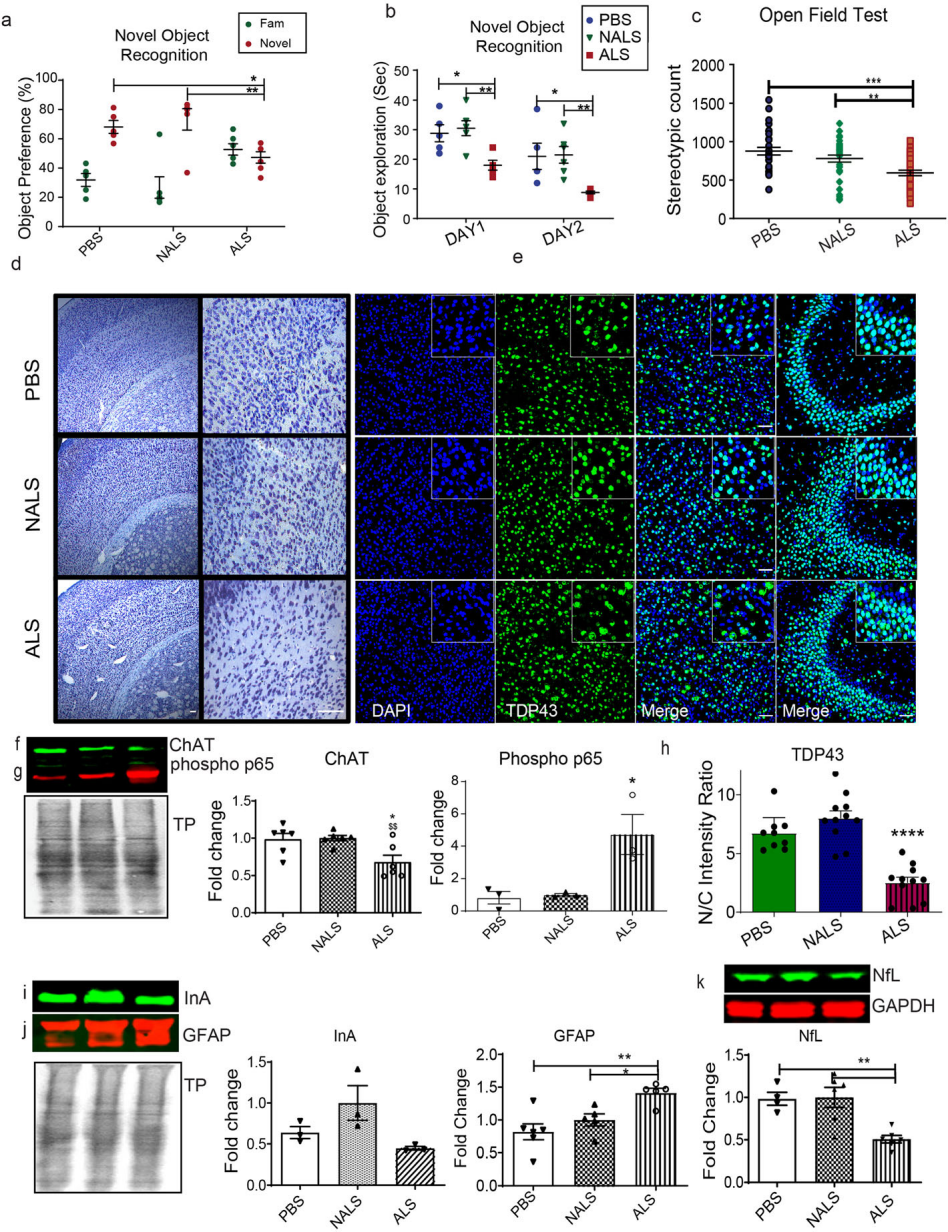
ALS-CSF triggered cognitive and pathological changes in the brain. (a, b) Novel object recognition. Graphs depicting object preference by mice between the familiar and novel object (a) and the overall time spent in exploring the objects (b) (n = 6). (c) Graph depicting stereotypic count from the OFT analysis. (n = 6). (d) Cresyl violet staining of cortical sections across the groups. (e.h) Cortical sections stained (e.) for DAPI (blue) and TDP43 (green), and quantification for the N/C intensity of TDP43 (h). Expression patterns and quantification of the immunoblots for ChAT (f), phospho p65 (g), InA (i) GFAP (j), and NfL (k.) proteins in the cortical and hippocampal lysates. (n = 3). Data are mean ± SEM. (*p ≤ 0.05, **p ≤ 0.01, and *** p ≤ 0.001) and fold changes are calculated compared to NALS. Scale bar = 50 μm

We used the novel object recognition test to monitor potential cognitive deficits triggered by exposure to ALS-CSF. The novel object recognition analysis revealed a diminished preference for the new object of hTDP43^WT^ mice administered with ALS-CSF when compared to the control groups (Fig. 5 a), suggesting memory impairment. Interestingly, the mice administered with ALS-CSF showed significantly reduced inclination towards the object exploration altogether (Fig. 5 b). Among the software generated data from the open field session, we also observed reduced stereotypic count in the ALS group as compared to the control groups (Fig. 5 c), further suggesting abnormal behavioral and cognitive patterns.

A qualitative histological analysis with cresyl violet performed on cortical and hippocampal sections (Fig. 5d.) revealed a reduction in the Nissl positive cells. Immunofluorescence microscopy of cortical and hippocampal sections revealed cytoplasmic TDP43 mislocalization in response to ALS-CSF (Fig. 5e) which was confirmed with a quantitative analysis demonstrating a decreased nuclear to cytoplasmic (N/C) TDP43 intensity ratio in the cortical sections from ALS group (Fig. 5 h). So, ALS-CSF infusion triggered TDP43 proteinopathy in neuronal subsets in the brain, in line with our observations with the spinal cord. Furthermore, ALS-CSF administration caused reduction of ChAT expression (Fig. 5f) as well as an inflammatory response involving enhanced phospho p65 (Fig. 5g) and GFAP (Fig. 5j) expression in brain tissue lysates from cortex and hippocampus. In the brain, ALS-CSF also induced a disorganization of neurofilaments with two-fold decrease in levels of NfLand InA (Fig. 5 i,k). These changes further demonstrate neuronal dysfunction and gliosis in the cortex and hippocampus of mice administered with ALS-CSF.

### Generation of NfL-RFP; hTDP43^WT^ double transgenic mice for neuronal translatome analysis

**Fig. 6:**
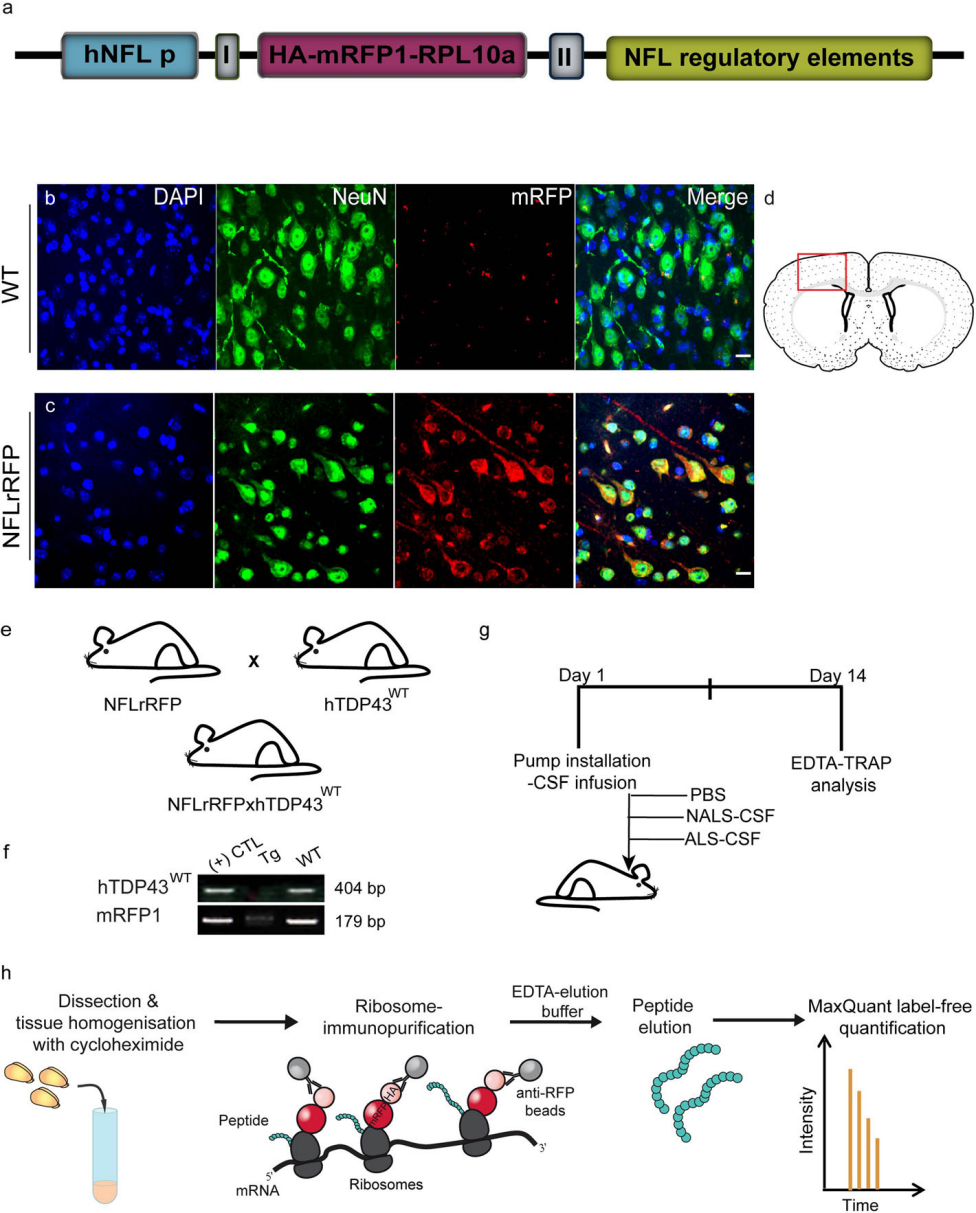
Characterization of the NfL-RFP; hTDP43WT double transgenic mice. (a) Schematic representation of HA-RFP1-tagged murine Rpl10a construction under control of the NFL promoter. —pBluescript KS +/− plasmid I, intervening sequence (IVS) and II, SV40 polyA. (b- d). Panel representing DAPI (blue), neuron specific marker, NeuN (green) and RFP (red) in the cortical sections (d) from the wild type (b) and NFL-rRFP (c) mice. (e- h) Scheme for the generation of double transgenic NfL-RFP; hTDP43WT mice (e). (f) represents the genotypic confirmation of the double transgene expression in the mice. (g) and (h) represent the scheme for the experimental procedure and extraction of peptides from the neuronal ribosomes using the EDTA-Trap method

To precisely delineate the impact of the toxic factors from ALS on the neuronal translational profiles, we took advantage of our modified translational affinity purification (EDTA-TRAP). Thus, a high affinity immunoprecipitation of translating ribosomes allows a pull down of cell-type specific proteomes. Briefly, collected ribosomes were subjected to EDTA elution buffer for nascent chains purification and the newly synthesized peptides were analyzed by mass spectrometry as described by Boutej and colleagues [6]. To do that, we generated a transgenic mouse model, named NFLrRFP, expressing the HA-mRFP1 tags fused to the N-terminus domain of the 60s ribosomal protein L10a (HA-mRFP1-RPL10a), under transcriptional control of the human NFL promoter (Fig. 6 a). As described in Charron et al., the human NFL promoter fragment contained sufficient elements to drive transgene expression in the nervous tissues of adult transgenic mice [9]. The transgenic mice are viable and do not develop overt phenotypes. We successfully validated by double immunofluorescence the restricted expression of HA-mRFP1-RPL10a transgene to neurons, by confirming the presence of the mRFP1 protein within the cortical neurons (Fig. 6 d), positive for the neuron specific marker, NeuN (Fig. 6 c), as well as its absence in cortical neurons from the wild-type mice (Fig. 6 b). Further, we generated the double transgenic mice NFLrRFP;hTDP43^WT^ by crossing the NFLrRFP transgenic mice with hTDP43^WT^ transgenic mice (Fig. 6 e). The presence of both genes was confirmed by genotyping the mouse DNA (Fig. 6 f). Finally, to investigate the neuronal translational patterns for our different study groups (Fig. 6 g), we performed a high-affinity immunoprecipitation assay from cortical and hippocampal homogenates to isolate the neuronal ribosomes and the peptides newly synthesized at these ribosomes (Fig. 6 h).

### Neuron-specific translational profile from the brain revealed dysregulation of multiple protein networks in response to ALS-CSF

**Fig. 7:**
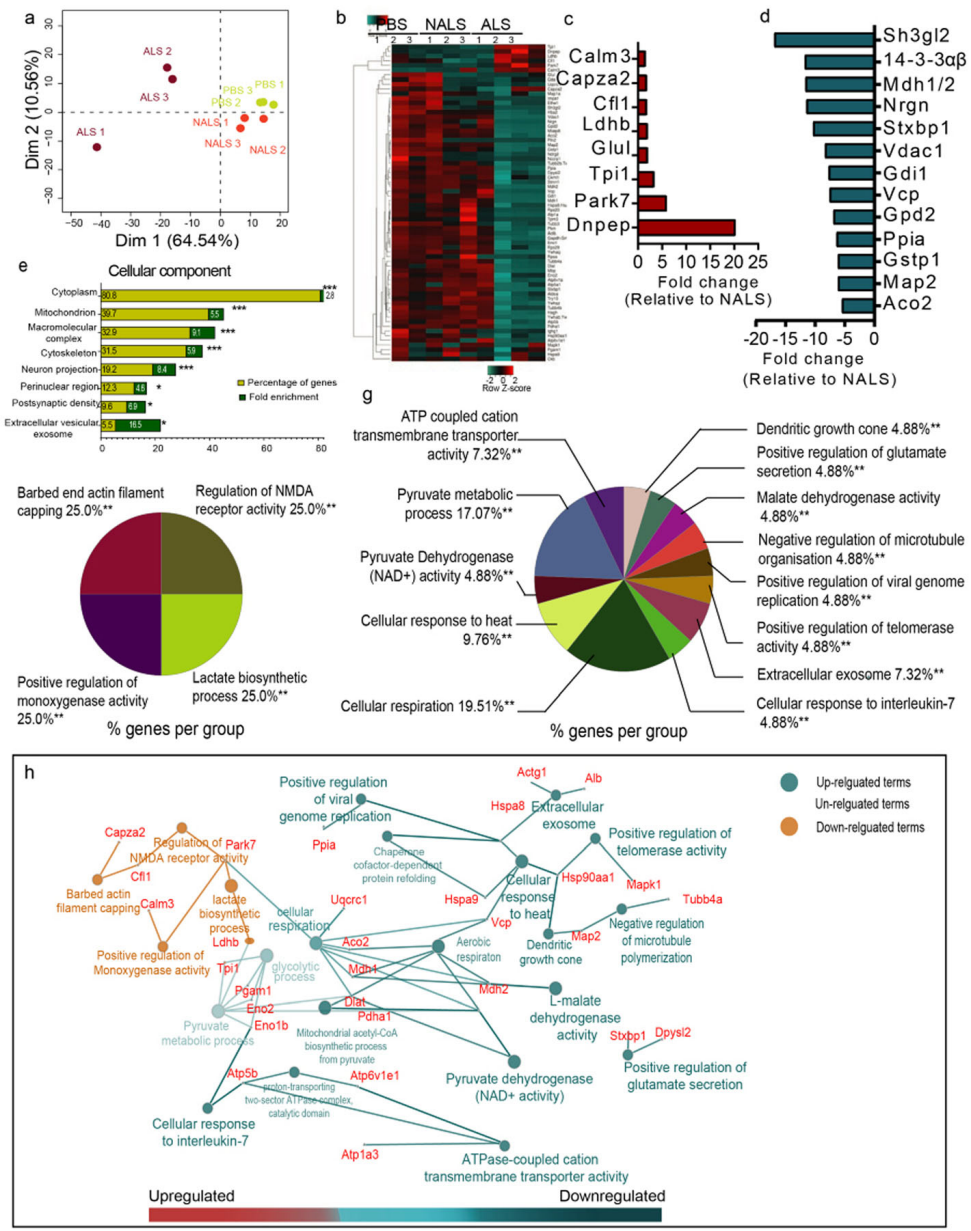
ALS-CSF altered the translational profiles of neurons. (a) Principal component analysis of the protein datasets for all the replicates across the groups. (b) Heatmap showing the pattern of regulation of the neuronal translatome across the groups. (c-d) Graph depicting fold changes (ALS vs NALS) in the prominently upregulated (red, c) and downregulated (blue, d) proteins. (e), Graph depicting the cellular (neuronal) components affected in response to ALS-CSF, when compared to NALS-CSF. (f, g). Pie charts describing the processes regulated by the proteins upregulated (f) and downregulated (g) in response to ALS-CSF (vs NALS-CSF). **p ≤ 0.01. (h) Overall network depicting the upregulated (red) as well as downregulated (blue) processes (ALS vs NALS). The analysis involved three biological replicates from NALS (n = 6) and ALS (n = 6) groups

To assess the impact of CSF exposure on the brain neuronal translatome, we immunoprecipitated neuronal ribosomes using the EDTA-TRAP approach [6] from cortical and hippocampal regions of NFLrRFPx hTDP43WT transgenic mice after 14 days i.c.v. infusion with PBS, NALS-CSF or ALS-CSF. The newly translated peptides bound to ribosomes were then analyzed by mass spectrometry. It is noteworthy that, although we started with the equal amount of tissue across all the groups, the concentration of protein extracts derived from the neuronal ribosomes of ALS-CSF group (0.36 ± 0.03 μg/μL) was consistently lower than that from the NALS group (1.56 ± 0.69 μg/μL). This likely reflects a block of mRNA translation associated with the pathology. Indeed, our analysis revealed that the neuronal synthesis of newly peptides was prominently downregulated in brain samples from ALS-CSF-administered mice when compared to PBS or NALS-CSF administered mice (Fig. 7).

Principal component analysis (PCA) revealed that the translational profile remained unaltered in the case of NALS infusion. However, infusion of ALS-CSF resulted in significant changes in the principal translation component of all the replicates within the ALS group (Fig. 7 a). A heat map was generated from the significantly altered proteins using hierarchical clustering and average linkage for all the groups. While the translational patterns remained relatable across the replicates from the PBS and NALS groups, significant variations were observed in the neuronal expression patterns from the mice administered with ALS-CSF (Fig. 7 b). Moreover, when the cluster analysis was performed with Cytoscape and ClueGo [5] for the GO biological and cellular terms corresponding to the dysregulated proteins (Supplementary Table S1), the multiple pathways significantly implicated in the ALS-CSF mediated insult converged to metabolic and structural dysfunction. While the upregulated proteins mainly affected the regulation of neurotransmitter activity through NMDA receptors, cell structure stabilization was, lactate biosynthesis and monooxygenase activity (Fig. 7 c, e), the downregulated peptides were involved in numerous metabolic processes including glucose and nucleotide metabolism. Additionally, heat shock proteins (HSPs), proteins involved in axonal growth, synaptic vesicular endocytosis, exosomal release, and glutamate secretion were also downregulated, hinting towards the potential of ALS-CSF to trigger metabolic stress, structural instability, neurotransmission, and synaptic dysfunction in the neurons (Fig. 7 d, f).

A clear distinction was observed in terms of the processes affected by either the upregulated (red) or the downregulated proteins (blue) (Fig. 7g). However, we observed an overlap at the terms ‘pyruvate metabolism’, ‘glycolysis’ and ‘cellular respiration’. Interestingly, all but one protein (TPI1) from the former two terms were downregulated. Translation of TPI1, a glycolytic enzyme lynchpin to many metabolic processes, including gluconeogenesis, fatty acid biosynthesis, and pentose phosphate pathway (PPP) [28], increased ~ 3 folds in the ALS-CSF group when compared to controls (Fig. 7 c). Another protein, Park7 was the only candidate upregulated in the term ‘cellular respiration’, which has been implicated in regulating cellular oxidative stress and well documented in ALS-CSF as well as the animal models for ALS [72, 73].

### ALS-CSF infusion altered cellular metabolic pathways and mitochondrial function

**Fig. 8:**
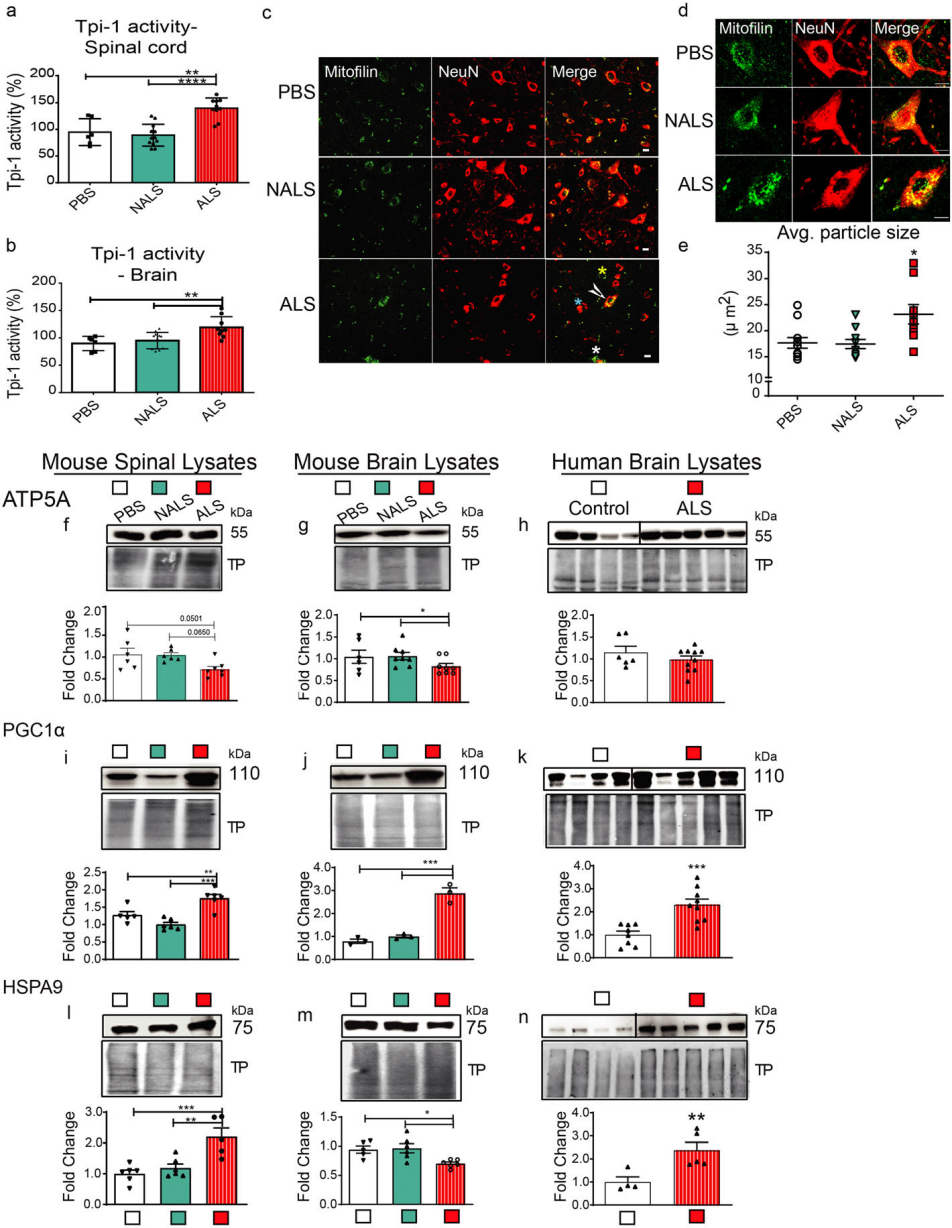
Metabolic and mitochondrial dysfunction were observed following ALS-CSF infusion. (a, b) Tpi-1 activity in mouse spinal cord (a) and brain (b) lysates (*n* = 5). (c-e) Mitochondrial aggregation. Panel (c) depicts spinal cord sections stained with the mitochondrial marker, mitofilin (green) and NeuN (red). Note the differences in the expression of mitofilin and NeuN within different neurons in the spinal cord from ALS mice (white arrowhead; white, green, and yellow asterisks). Panel (d) represents the patterns of mitochondrial localization within the neurons. (e) denotes the analysis of particle size for the mitofilin staining. (n = 3) Scale Bar = 20 μm. (f-h) Expression patterns and quantification of the immunoblots for ATP5A, from mouse spinal cord (f) (n = 3), and brain (g) (n = 3), as well as human cortical (h) lysates (*n* = 4, controls; n = 5, ALS). (i.-k.) Expression patterns and quantification of the immunoblots for PGC1α, from mouse spinal cord (i) (n = 3), and brain (j) (n = 3), as well as human cortical(k) lysates (n = 4, controls; n = 5, ALS). (l-n) Expression patterns and quantification of the immunoblots for HSP9A, from mouse spinal cord (l) (n = 3), and brain (m) (n = 3), as well as human cortical(n) lysates. (n = 4, controls; n = 5, ALS). Data are mean ± SEM. (*p ≤ 0.05, **p ≤ 0.01, and *** p ≤ 0.001)

The translational changes in response to ALS-CSF strongly advocated a metabolic involvement in ALS pathology. This prompted us to investigate the metabolic load in-vivo. In the EDTA-TRAP analysis, we found a three-fold upregulation in the translation of Tpi1 (Table S1). Therefore, we compared the levels of Tpi1 activity across the groups and observed a significant upregulation of Tpi1 activity in the brain (cortex and hippocampus) and spinal cord of hTDP43^WT^ transgenic mice in response to ALS-CSF (Fig. 8 a, b). This provided validation for a functional dysfunction of pyruvate metabolism and led us to investigate the downstream processes in the energy metabolism pathways. Immunohistochemical investigations using the mitochondrial marker, mitofilin, revealed substantially divergent mitochondrial distribution patterns in response to ALS-CSF. Interestingly, inter-neuronal differences were also seen well within spinal neuronal population in the ALS-CSF group. While some neurons stained intensely for NeuN as well as mitofilin (Fig. 8 c, white arrowhead), others displayed intense NeuN, but weak mitofilin immunoreactivity (Fig. 8 c, blue asterisk). The third set of neurons displayed intense mitofilin but weak NeuN immunoreactivity (Fig. 8 c, white asterisk). Incidentally, a few cells corresponding to neuronal size lacked NeuN expression but stained positive to mitofilin (Fig. 8 c, yellow asterisk). The extent of NeuN immunoreactivity may provide a window into the status of neuronal integrity. Concomitantly, differential expression of mitofilin in different neurons might indicate the extent of metabolic stress in the dying neurons as well as a failed attempt at metabolic compensation by the active neurons leading to a metabolic overload.

Further, quantification of the average particle size of mitofilin using particle analysis feature of ImageJ revealed significantly increased particle size, indicating abnormal mitochondrial cluttering or aggregation in the neurons in response to ALS-CSF (Fig. 8 d, e). However, we did not observe any significant change in the overall expression of mitofilin across the groups (unpublished observations). To access the functional integrity of mitochondria, we also investigated the expression patterns of ATP5A and found it to be significantly downregulated in the brain lysates from the ALS-CSF group (Fig. 8 g). Although not significant, a trend of downregulation was also observed in the spinal cords (Fig. 8 f). We also checked its expression in the human cortical lysates, but observed no significant change, possibly owing to the large variations observed within the dataset (Fig. 8 h).

Dysregulated mitochondrial physiology and induction of pathology led us to probe PGC1α, the transcriptional coactivator central to the mitochondrial homeostasis [61]. Intriguingly, we observed a tremendous upregulation of the protein levels in the spinal cord (Fig. 8 i), and the brain (Fig. 8 j) lysates from the mice subjected to ALS-CSF, but also in the cortical lysates from the ALS patients (Fig. 8 k).

Heat shock response, another significantly affected process in our neuronal translatome analysis, led us to converge to HSPA9 (mtHSP70 protein/mortalin/GRP75), a 75kd mitochondrial HSP involved in various pathways including chaperoning, neural signaling and apoptosis [23] . Notably, HSPA9 expression was upregulated in the spinal cord samples from the mice administered with ALS-CSF (Fig. 8l) as well as in the human brain from ALS (Fig. 8n).

## Discussion

The results presented here support the hypothesis that the CSF is a factor that may be involved in the spreading of ALS pathogenesis [57]. Thus, chronic i.c.v. infusion of pooled CSF samples from sporadic ALS patients into transgenic hTDP43^WT^ mice triggered ALS-like phenotypes and pathology including the formation of cytoplasmic TDP43 aggregates and TDP43 phosphorylated species. Interestingly, the use of a recipient mouse expressing human TDP43 was a requirement to trigger TDP43 pathology in motor neurons via i.c.v. infusion of ALS-CSF. No cytoplasmic TDP43 accumulations were observed in normal 8-month-old mice administered ALS-CSF i.c.v. for 14 days (Supplementary Fig. S1 a, b) even though we did observe motor phenotypes in response to ALS-CSF with normal mice albeit the magnitude was less pronounced than with hTDP43 transgenic mice. So, the presence of hTDP43 can be viewed as factor that enhances response to ALS-CSF toxicity and a susceptibility factor for the occurrence of TDP43 pathology. Moreover, there is likely a dose-threshold for the seeding of cytoplasmic TDP43 aggregation that was facilitated by the extra TDP43 levels in the transgenic mouse model. This would be consistent with the concept of a prion-like mechanism involving human TDP43 species in the CSF from ALS patients in the transmission of TDP43 proteinopathy, as suggested previously based on cultured cells exposed to exosomes from the brain of ALS cases [32]. It is noteworthy that aging is a susceptibility factor in this mouse paradigm. Unlike 8-months-old hTDP43^WT^ mice, young recipient hTDP43^WT^ mice at 3 to 5 months of age did not exhibit behavioral phenotypes and atrophy of muscle fibers after 2-weeks infusion of ALS-CSF (Supplementary Fig. S3).

Inflammation is another factor that may exacerbate TDP43 pathology [13]. Infusion of ALS-CSF in hTDP43 transgenic mice resulted in gliosis. Interestingly, we observed a robust upregulation of galectin-3, a microglial protein that has been shown to correlate with disease progression in ALS-CSF [37] and which is a candidate CSF bio-marker for ALS [75]. Our results showed a concomitant decrease in arginase levels suggesting that gliosis was skewed towards a pro-inflammatory phenotype after ALS-CSF administration. There was also an upregulation of inflammation markers Chit-1 and phospho-p65 NF-κB in ALS-CSF groups compared to the PBS group. However, the upregulation of these proteins in the NALS-CSF group as well possibly indicates their general involvement in response to CSF [22, 57].

The i.c.v. infusion of ALS-CSF caused dramatic changes in levels of IF proteins with severe downregulation of neurofilament proteins (NfL, NfM, and InA) and upregulation of type III IF proteins peripherin and GFAP. Previous transgenic mouse studies demonstrated that neurofilament disorganization can cause formation of neurofilament aggregates, axonal transport defects and degeneration of motor neurons [3, 14, 20, 34, 38, 71] In addition, NfL deficits can cause synaptic dysfunction with reduction of dendritic spines and GluN1 levels [74]. An overexpression of peripherin transgene in mice in the context of NfL deficit, as observed here in hTDP43 mice infused with ALS-CSF, was found to provoke the formation of IF aggregates, axonal transport defects and motor neuron death [2, 42].

How exposure to ALS-CSF can affect levels of IF proteins remains to be investigated. Yet, it is well established that TDP43 can bind to neurofilament mRNAs and other mRNAs of cytoskeletal proteins affected in the present study [16, 60]. Accordingly, it is conceivable that excess cytoplasmic TDP43 binding to neurofilament mRNAs could repress translation resulting in reduced synthesis of neurofilament proteins. On the other hand, upregulation of neuronal peripherin after ALS-CSF infusion may be due in part to the upregulation of peripherin gene transcription by interleukin-6 [58] an inflammatory cytokine released during gliosis as a response to ALS-CSF [44, 45].

The neuron-specific translational profiles revealed the dysregulation of multiple protein networks in response to ALS-CSF. The ALS-CSF exposure caused downregulation of several cytoskeletal proteins associated with microtubules and actin filaments, proteins involved in trafficking of vesicles and exosomal release, HSPs, proteins involved in axonal growth and glutamate secretion. Such changes are compatible with neurodegenerative events occurring in ALS [27, 35, 43]. Moreover, the ALS-CSF exposure triggered alterations in aerobic respiration and metabolic pathways of relevance to current knowledge of ALS pathology. Metabolic abnormalities in neurodegeneration have increasingly gained attention to partially explain energetic stress and selective vulnerability in ALS [66]. The altered metabolic processes due to ALS-CSF in the present study reflect a steep deviation from a balanced neuronal metabolic state. On the one hand, increased Tpi1 activity in response to ALS-CSF may indicate a compensatory mechanism to mitigate the neuronal energy crisis following diminished aerobic respiration, including downregulated ATP5A translation/expression observed in the present study. On the other hand, the upregulation in Tpi1 activity can further aggravate the pathology, since its inhibition is known to confer oxidative stress tolerance [29]. Indeed, a concomitant upregulation in LDH-b synthesis in the present study, along with the disrupted glycolysis and downstream pathways further indicate disbalance and formation of alternate metabolites, favoring toxicity. Upregulation of the glycolytic pathway, coupled with a reduction in ATP generation have also been discussed in the pathology of depression and hypoxia as compensatory mechanisms [1, 19]. More recently, increased glycolysis has been discussed as a compensatory mechanism in a Drosophila model of ALS overexpressing TDP43 [41]. The astroglial activation and glutamatergic disbalance may further aggravate the insult by overstimulating glycolysis and lactate production through the metabolic coupling.

Our results demonstrate that ALS-CSF infusion caused mitochondrial dysfunction in agreement with a previous study [55], adding mitochondrial aggregation and altered homeostasis to the repertoire. Mitochondrial aggregation and dysregulated HSPA9 levels indicate neuronal apoptosis, as well as HSPA9-mediated dysregulation in the mitochondrial-ER coupling, protein trafficking, organellar proteostasis and sensitivity to glutamate excitotoxicity [23, 29, 30]. Concomitantly, the upregulation of PGC1α in response to ALS-CSF infusion indicates dysregulated mitochondrial turnover through a disbalance between mitophagy and mitogenesis [65]. The combined results introduce metabolic dysfunction as a prominent feature of neuronal pathology seen in response to ALS-CSF and warrant future, detailed investigations in the mechanism of mitochondrial involvement and energy homeostasis in ALS pathogenesis.

Our finding that CSF samples from sporadic ALS patients contain toxic factors that can transmit the disease to an appropriate mouse model opens new research avenues on the discovery of new therapeutic targets. The transmission of toxic TDP43 species from the CSF, free or within exosomes, is a possibility that should be prioritized in future studies in the light of previous report that exosomes containing TDP43 from the brain of ALS cases were able to transmit aggregation-prone TDP43 to cultured cells [32]. Many other components of the CSF might also contribute in part to propagation of pathogenesis and inflammation. The complexity of CSF will likely require serial fractionation of CSF to identify proteins or RNA species that contribute to pathological changes in the cultured cell system and to the in-vivo mouse model described here.

The hTDP43 transgenic mouse as the recipient of CSF infusion should provide a unique mouse model system for testing therapeutics aiming to target TDP43 pathology and/or other pathogenic pathways in the context of sporadic ALS disease. Future studies with this mouse model are needed to determine whether distinct pathological or molecular signatures might arise from the CSF of ALS patients with slow or fast progressive disease. In any case, this mouse model system can be used to quickly test potential drugs aiming to neutralize the toxicity of CSF samples from ALS patients. Finally, despite robust phenotypes and pathological changes triggered ALS-CSF infusion, the disease did not evolve to mouse paralysis and death over the 14-day period. Future studies are needed to further examine the effects of longer time CSF administration on disease severity and to assess the effects of cessation of CSF infusion on disease progression.

## Supplementary information

**Additional file 1: Supplementary Figure S1.** No significant cytoplasmic mislocalization of TDP43 in normal mice infused with ALS-CSF. **Supplementary Figure S2.** Normal mice are less affected than hTDP43 transgenic mice to motor dysfunction and muscle damage induced by ALS-CSF infusion. **Supplementary Figure S3.** Age-dependent pathology in the hTDP-43 transgenic mice infused with ALS-CSF. **Table S1.** List of neuronal peptides with significantly altered translational pattern in response to ALS-CSF.

## Data Availability

Data are available on reasonable request, from the first and corresponding authors.

## References

[1] Agbor TA, Cheong A, Comerford KM, Scholz CC, Bruning U, Clarke A, Cummins EP, Cagney G, Taylor CT (2011). Small ubiquitin-related modifier (SUMO)-1 promotes glycolysis in hypoxia. J Biol Chem.

[2] Beaulieu J-M, Nguyen MD, Julien J-P (1999). Late onset death of motor neurons in mice overexpressing wild-type peripherin. J Cell Biol.

[3] Beaulieu JM, Jacomy H, Julien JP (2000). Formation of intermediate filament protein aggregates with disparate effects in two transgenic mouse models lacking the neurofilament light subunit. J Neurosci.

[4] Berning BA, Walker AK (2019). The pathobiology of TDP-43 C-terminal fragments in ALS and FTLD. Front Neurosci.

[5] Bindea G, Mlecnik B, Hackl H, Charoentong P, Tosolini M, Kirilovsky A, Fridman WH, Pages F, Trajanoski Z, Galon J (2009). ClueGO: a Cytoscape plug-in to decipher functionally grouped gene ontology and pathway annotation networks. Bioinformatics.

[6] Boutej H, Rahimian R, Thammisetty SS, Beland LC, Lalancette-Hebert M, Kriz J (2017). Diverging mRNA and protein networks in activated microglia reveal SRSF3 suppresses translation of highly Upregulated innate immune transcripts. Cell Rep.

[7] Brooks BR, Miller RG, Swash M, Munsat TL, World Federation of Neurology Research Group on Motor Neuron D (2000). El Escorial revisited: revised criteria for the diagnosis of amyotrophic lateral sclerosis. Amyotroph Lateral Scler Other Motor Neuron Disord.

[8] Cady J, Allred P, Bali T, Pestronk A, Goate A, Miller TM, Mitra RD, Ravits J, Harms MB, Baloh RH (2015). Amyotrophic lateral sclerosis onset is influenced by the burden of rare variants in known amyotrophic lateral sclerosis genes. Ann Neurol.

[9] Charron G, Guy LG, Bazinet M, Julien JP (1995). Multiple neuron-specific enhancers in the gene coding for the human neurofilament light chain. J Biol Chem.

[10] Chio A, Benzi G, Dossena M, Mutani R, Mora G (2005). Severely increased risk of amyotrophic lateral sclerosis among Italian professional football players. Brain.

[11] Chu JF, Majumder P, Chatterjee B, Huang SL, Shen CJ (2019). TDP-43 regulates coupled dendritic mRNA transport-translation processes in co-operation with FMRP and Staufen1. Cell Rep.

[12] Corbo M, Hays AP (1992). Peripherin and neurofilament protein coexist in spinal spheroids of motor neuron disease. J Neuropathol Exp Neurol.

[13] Correia AS, Patel P, Dutta K, Julien JP (2015). Inflammation induces TDP-43 Mislocalization and aggregation. PLoS One.

[14] Cote F, Collard JF, Julien JP (1993). Progressive neuronopathy in transgenic mice expressing the human neurofilament heavy gene: a mouse model of amyotrophic lateral sclerosis. Cell.

[15] Cox J, Mann M (2008). MaxQuant enables high peptide identification rates, individualized ppb-range mass accuracies and proteome-wide protein quantification. Nat Biotechnol.

[16] Coyne AN, Siddegowda BB, Estes PS, Johannesmeyer J, Kovalik T, Daniel SG, Pearson A, Bowser R, Zarnescu DC (2014). Futsch/MAP 1B mRNA is a translational target of TDP-43 and is neuroprotective in a Drosophila model of amyotrophic lateral sclerosis. J Neurosci.

[17] Cykowski MD, Powell SZ, Appel JW, Arumanayagam AS, Rivera AL, Appel SH (2018). Phosphorylated TDP-43 (pTDP-43) aggregates in the axial skeletal muscle of patients with sporadic and familial amyotrophic lateral sclerosis. Acta Neuropathol Commun.

[18] Das K, Nag C, Ghosh M (2012). Familial, environmental, and occupational risk factors in development of amyotrophic lateral sclerosis. N Am J Med Sci.

[19] Detka J, Kurek A, Kucharczyk M, Glombik K, Basta-Kaim A, Kubera M, Lason W, Budziszewska B (2015). Brain glucose metabolism in an animal model of depression. Neuroscience.

[20] Didonna A, Opal P (2019). The role of neurofilament aggregation in neurodegeneration: lessons from rare inherited neurological disorders. Mol Neurodegener.

[21] Feneberg E, Gray E, Ansorge O, Talbot K, Turner MR (2018). Towards a TDP-43-based biomarker for ALS and FTLD. Mol Neurobiol.

[22] Filer A, Bik M, Parsonage GN, Fitton J, Trebilcock E, Howlett K, Cook M, Raza K, Simmons DL, Thomas AM (2009). Galectin 3 induces a distinctive pattern of cytokine and chemokine production in rheumatoid synovial fibroblasts via selective signaling pathways. Arthritis Rheum.

[23] Flachbartova Z, Kovacech B (2013). Mortalin - a multipotent chaperone regulating cellular processes ranging from viral infection to neurodegeneration. Acta Virol.

[24] Frakes AE, Ferraiuolo L, Haidet-Phillips AM, Schmelzer L, Braun L, Miranda CJ, Ladner KJ, Bevan AK, Foust KD, Godbout JP (2014). Microglia induce motor neuron death via the classical NF-kappaB pathway in amyotrophic lateral sclerosis. Neuron.

[25] Gille B, De Schaepdryver M, Dedeene L, Goossens J, Claeys KG, Van Den Bosch L, Tournoy J, Van Damme P, Poesen K (2019). Inflammatory markers in cerebrospinal fluid: independent prognostic biomarkers in amyotrophic lateral sclerosis?. J Neurol Neurosurg Psychiatry.

[26] Gomez-Pinedo U, Galan L, Yanez M, Matias-Guiu J, Valencia C, Guerrero-Sola A, Lopez-Sosa F, Brin JR, Benito-Martin MS, Leon-Espinosa G (2018). Histological changes in the rat brain and spinal cord following prolonged intracerebroventricular infusion of cerebrospinal fluid from amyotrophic lateral sclerosis patients are similar to those caused by the disease. Neurologia.

[27] Grossman M (2019). Amyotrophic lateral sclerosis - a multisystem neurodegenerative disorder. Nat Rev Neurol.

[28] Gruning NM, Du D, Keller MA, Luisi BF, Ralser M (2014). Inhibition of triosephosphate isomerase by phosphoenolpyruvate in the feedback-regulation of glycolysis. Open Biol.

[29] Haga N, Fujita N, Tsuruo T (2003). Mitochondrial aggregation precedes cytochrome c release from mitochondria during apoptosis. Oncogene.

[30] Honrath B, Metz I, Bendridi N, Rieusset J, Culmsee C, Dolga AM (2017). Glucose-regulated protein 75 determines ER-mitochondrial coupling and sensitivity to oxidative stress in neuronal cells. Cell Death Discov.

[31] Hooten KG, Beers DR, Zhao W, Appel SH (2015). Protective and toxic Neuroinflammation in amyotrophic lateral sclerosis. Neurotherapeutics.

[32] Iguchi Y, Eid L, Parent M, Soucy G, Bareil C, Riku Y, Kawai K, Takagi S, Yoshida M, Katsuno M (2016). Exosome secretion is a key pathway for clearance of pathological TDP-43. Brain.

[33] Ingre C, Roos PM, Piehl F, Kamel F, Fang F (2015). Risk factors for amyotrophic lateral sclerosis. Clin Epidemiol.

[34] Julien J-P, Mushynski WE, Moldave K (1998). Neurofilaments in health and disease. Progress in nucleic acid research and molecular biology.

[35] Kiernan MC, Vucic S, Cheah BC, Turner MR, Eisen A, Hardiman O, Burrell JR, Zoing MC (2011). Amyotrophic lateral sclerosis. Lancet.

[36] Kunst CB (2004). Complex genetics of amyotrophic lateral sclerosis. Am J Hum Genet.

[37] Lalancette-Hebert M, Swarup V, Beaulieu JM, Bohacek I, Abdelhamid E, Weng YC, Sato S, Kriz J (2012). Galectin-3 is required for resident microglia activation and proliferation in response to ischemic injury. J Neurosci.

[38] Lee MK, Marszalek JR, Cleveland DW (1994). A mutant neurofilament subunit causes massive, selective motor neuron death: implications for the pathogenesis of human motor neuron disease. Neuron.

[39] Liu EY, Russ J, Cali CP, Phan JM, Amlie-Wolf A, Lee EB (2019). Loss of nuclear TDP-43 is associated with Decondensation of LINE Retrotransposons. Cell Rep.

[40] Lomen-Hoerth C, Anderson T, Miller B (2002). The overlap of amyotrophic lateral sclerosis and frontotemporal dementia. Neurology.

[41] Manzo E, Lorenzini I, Barrameda D, O’Conner AG, Barrows JM, Starr A, Kovalik T, Rabichow BE, Lehmkuhl EM, Shreiner DD et al (2019) Glycolysis upregulation is neuroprotective as a compensatory mechanism in ALS. Elife 8. 10.7554/eLife.45114 10.7554/eLife.45114 PMC6557627 31180318

[42] Millecamps S, Gowing G, Corti O, Mallet J, Julien JP (2007). Conditional NF-L transgene expression in mice for in vivo analysis of turnover and transport rate of neurofilaments. J Neurosci.

[43] Mishra P-S, Singh A (2019). Heat shock proteins in neural signaling: implications in health and disease.

[44] Mishra PS, Dhull DK, Nalini A, Vijayalakshmi K, Sathyaprabha TN, Alladi PA, Raju TR (2016). Astroglia acquires a toxic neuroinflammatory role in response to the cerebrospinal fluid from amyotrophic lateral sclerosis patients. J Neuroinflammation.

[45] Mishra PS, Vijayalakshmi K, Nalini A, Sathyaprabha TN, Kramer BW, Alladi PA, Raju TR (2017). Etiogenic factors present in the cerebrospinal fluid from amyotrophic lateral sclerosis patients induce predominantly pro-inflammatory responses in microglia. J Neuroinflammation.

[46] Mitchell JC, Constable R, So E, Vance C, Scotter E, Glover L, Hortobagyi T, Arnold ES, Ling SC, McAlonis M (2015). Wild type human TDP-43 potentiates ALS-linked mutant TDP-43 driven progressive motor and cortical neuron degeneration with pathological features of ALS. Acta Neuropathol Commun.

[47] Picher-Martel V, Renaud L, Bareil C, Julien J-P (2019). Neuronal expression of UBQLN2 P497H exacerbates TDP-43 pathology in TDP-43 G348C mice through interaction with ubiquitin. Mol Neurobiol.

[48] Picher-Martel V, Valdmanis PN, Gould PV, Julien JP, Dupre N (2016). From animal models to human disease: a genetic approach for personalized medicine in ALS. Acta Neuropathol Commun.

[49] Pozzi S, Thammisetty SS, Codron P, Rahimian R, Plourde KV, Soucy G, Bareil C, Phaneuf D, Kriz J, Gravel C (2019). Virus-mediated delivery of antibody targeting TAR DNA-binding protein-43 mitigates associated neuropathology. J Clin Invest.

[50] Rao MS, Devi MG, Nalini A, Shahani N, Raju TR (1995). Neurofilament phosphorylation is increased in ventral horn neurons of neonatal rat spinal cord exposed to cerebrospinal fluid from patients with amyotrophic lateral sclerosis. Neurodegeneration.

[51] Ratti A, Buratti E (2016). Physiological functions and pathobiology of TDP-43 and FUS/TLS proteins. J Neurochem.

[52] Sabitha KR, Sanjay D, Savita B, Raju TR, Laxmi TR (2016). Electrophysiological characterization of Nsc-34 cell line using microelectrode Array. J Neurol Sci.

[53] Sankaranarayani R, Nalini A, Rao Laxmi T, Raju TR (2010). Altered neuronal activities in the motor cortex with impaired motor performance in adult rats observed after infusion of cerebrospinal fluid from amyotrophic lateral sclerosis patients. Behav Brain Res.

[54] Shanmukha S, Narayanappa G, Nalini A, Alladi PA, Raju TR (2018). Sporadic amyotrophic lateral sclerosis (SALS) - skeletal muscle response to cerebrospinal fluid from SALS patients in a rat model. Dis Model Mech.

[55] Sharma A, Varghese AM, Vijaylakshmi K, Sumitha R, Prasanna VK, Shruthi S, Chandrasekhar Sagar BK, Datta KK, Gowda H, Nalini A (2016). Cerebrospinal fluid from sporadic amyotrophic lateral sclerosis patients induces mitochondrial and Lysosomal dysfunction. Neurochem Res.

[56] Shi P, Gal J, Kwinter DM, Liu X, Zhu H (2010). Mitochondrial dysfunction in amyotrophic lateral sclerosis. Biochim Biophys Acta.

[57] Smith R, Myers K, Ravits J, Bowser R (2015). Amyotrophic lateral sclerosis: is the spinal fluid pathway involved in seeding and spread?. Med Hypotheses.

[58] Sterneck E, Kaplan DR, Johnson PF (1996). Interleukin-6 induces expression of peripherin and cooperates with Trk receptor signaling to promote neuronal differentiation in PC12 cells. J Neurochem.

[59] Strong MJ (2008). The syndromes of frontotemporal dysfunction in amyotrophic lateral sclerosis. Amyotrophic Lateral Sclerosis.

[60] Strong MJ, Volkening K, Hammond R, Yang W, Strong W, Leystra-Lantz C, Shoesmith C (2007). TDP43 is a human low molecular weight neurofilament (hNFL) mRNA-binding protein. Mol Cell Neurosci.

[61] Summermatter S, Santos G, Perez-Schindler J, Handschin C (2013). Skeletal muscle PGC-1alpha controls whole-body lactate homeostasis through estrogen-related receptor alpha-dependent activation of LDH B and repression of LDH a. Proc Natl Acad Sci U S A.

[62] Tatem KS, Quinn JL, Phadke A, Yu Q, Gordish-Dressman H, Nagaraju K (2014) Behavioral and locomotor measurements using an open field activity monitoring system for skeletal muscle diseases. J Vis Exp 51785. 10.3791/51785 10.3791/51785 PMC4672952 25286313

[63] Thompson AG, Gray E, Bampton A, Raciborska D, Talbot K, Turner MR (2019). CSF chitinase proteins in amyotrophic lateral sclerosis. J Neurol Neurosurg Psychiatry.

[64] Turner MR, Al-Chalabi A, Chio A, Hardiman O, Kiernan MC, Rohrer JD, Rowe J, Seeley W, Talbot K (2017). Genetic screening in sporadic ALS and FTD. J Neurol Neurosurg Psychiatry.

[65] Vainshtein A, Desjardins EM, Armani A, Sandri M, Hood DA (2015). PGC-1alpha modulates denervation-induced mitophagy in skeletal muscle. Skelet Muscle.

[66] Vandoorne T, De Bock K, Van Den Bosch L (2018). Energy metabolism in ALS: an underappreciated opportunity?. Acta Neuropathol.

[67] Varghese AM, Sharma A, Mishra P, Vijayalakshmi K, Harsha HC, Sathyaprabha TN, Bharath SM, Nalini A, Alladi PA, Raju TR (2013). Chitotriosidase - a putative biomarker for sporadic amyotrophic lateral sclerosis. Clin Proteomics.

[68] Vijayalakshmi K, Alladi PA, Sathyaprabha TN, Subramaniam JR, Nalini A, Raju TR (2009). Cerebrospinal fluid from sporadic amyotrophic lateral sclerosis patients induces degeneration of a cultured motor neuron cell line. Brain Res.

[69] Vijayalakshmi K, Ostwal P, Sumitha R, Shruthi S, Varghese AM, Mishra P, Manohari SG, Sagar BC, Sathyaprabha TN, Nalini Aet al (2015) Role of VEGF and VEGFR2 receptor in reversal of ALS-CSF induced degeneration of NSC-34 motor neuron cell line. Mol Neurobiol 51: 995–1007 Doi 10.1007/s12035-014-8757-y 10.1007/s12035-014-8757-y 24880751

[70] Wang C, Yue F, Kuang S (2017) Muscle histology characterization using H&E Staining and muscle Fiber type classification using immunofluorescence staining. Bio Protoc 7: Doi 10.21769/BioProtoc.2279 10.21769/BioProtoc.2279 PMC5526362 28752107

[71] Wong NK, He BP, Strong MJ (2000). Characterization of neuronal intermediate filament protein expression in cervical spinal motor neurons in sporadic amyotrophic lateral sclerosis (ALS). J Neuropathol Exp Neurol.

[72] Xu X, Martin F, Friedman JS (2010). The familial Parkinson's disease gene DJ-1 (PARK7) is expressed in red cells and plays a role in protection against oxidative damage. Blood Cells Mol Dis.

[73] Yamashita S, Mori A, Kimura E, Mita S, Maeda Y, Hirano T, Uchino M (2010). DJ-1 forms complexes with mutant SOD1 and ameliorates its toxicity. J Neurochem.

[74] Yuan A, Veeranna, Sershen H, Basavarajappa BS, Smiley JF, Hashim A, Bleiwas C, Berg M, Guifoyle DN, Subbanna S (2018). Neurofilament light interaction with GluN1 modulates neurotransmission and schizophrenia-associated behaviors. Transl Psychiatry.

[75] Zhou JY, Afjehi-Sadat L, Asress S, Duong DM, Cudkowicz M, Glass JD, Peng J (2010). Galectin-3 is a candidate biomarker for amyotrophic lateral sclerosis: discovery by a proteomics approach. J Proteome Res.

