# Supplementary material for: Transmission of ALS pathogenesis by the cerebrospinal fluid
Source: Acta Neuropathol Commun. 2020 May 7;8:65. doi: 10.1186/s40478-020-00943-4 (PMC7206749; doi:10.1186/s40478-020-00943-4)
Supplement: Supplementary file 1 — Additional file 1: Supplementary Figure S1. No significant cytoplasmic mislocalization of TDP43 in normal mice infused with ALS-CSF. Supplementary Figure S2. Normal mice are less affected than hTDP43 transgenic mice to motor dysfunction and muscle damage induced by ALS-CSF infusion. Supplementary Figure S3. Age-dependent pathology in the hTDP-43 transgenic mice infused with ALS-CSF. Table S1. List of neuronal peptides with significantly altered translational pattern in response to ALS-CSF. [file 40478_2020_943_MOESM1_ESM.docx]

**Supplemental information**

**Transmission of ALS pathogenesis by the cerebrospinal fluid**

Pooja Shree Mishra, Hejer Boutej, Geneviève Soucy, Christine Bareil, Sunny Kumar, Vincent Picher-Martel, Nicholas Dupré, Jasna Kriz, and Jean-Pierre Julien


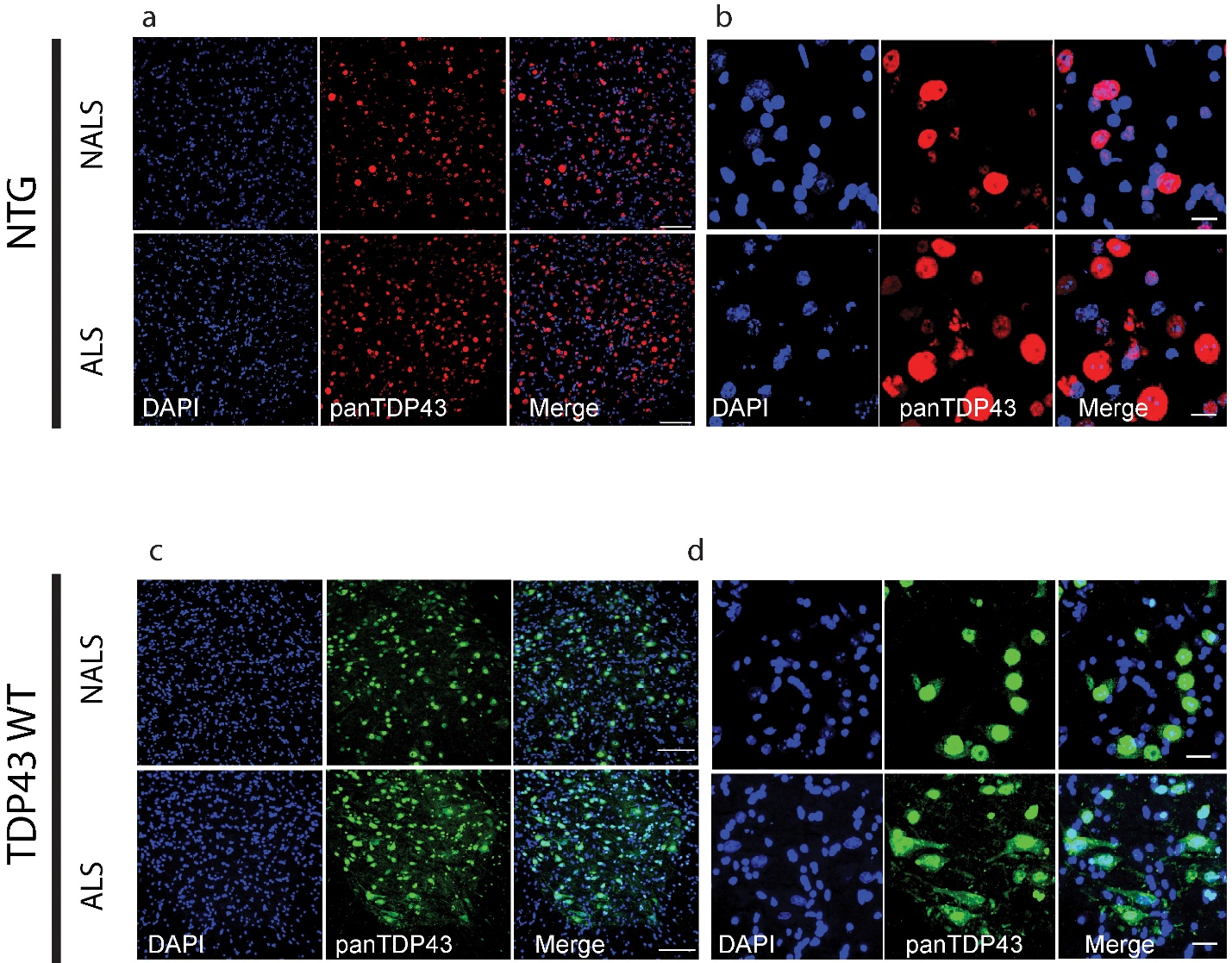


**Fig. S1. No significant cytoplasmic mislocalization of TDP43 in normal mice infused with ALS-CSF.**

(a, b) TDP43 localization in spinal cord sections of non-transgenic mice (8 months of age) infused with NALS-CSF and ALS-CSF (Scale bar= 50 µm). Note the absence of cytoplasmic TDP43 accumulations after ALS-CSF treatment. (c,d) TDP43 localization in spinal cord sections from TDP43^WT^ transgenic mice (8 months of age) infused with NALS-CSF and ALS-CSF (Scale bar= 50 µm). ALS-CSF induced cytoplasmic TDP-43 aggregates. The rabbit anti-TDP43 (panTDP43, proteintech #10782) used for the study was fluorescently lableled with anti-rabbit Alexa-568 (red) secondary antibody for NTG mice (a, b) and anti-rabbit Alexa-488 secondary antibody (green) for  the TDP43WT mice (c,d).


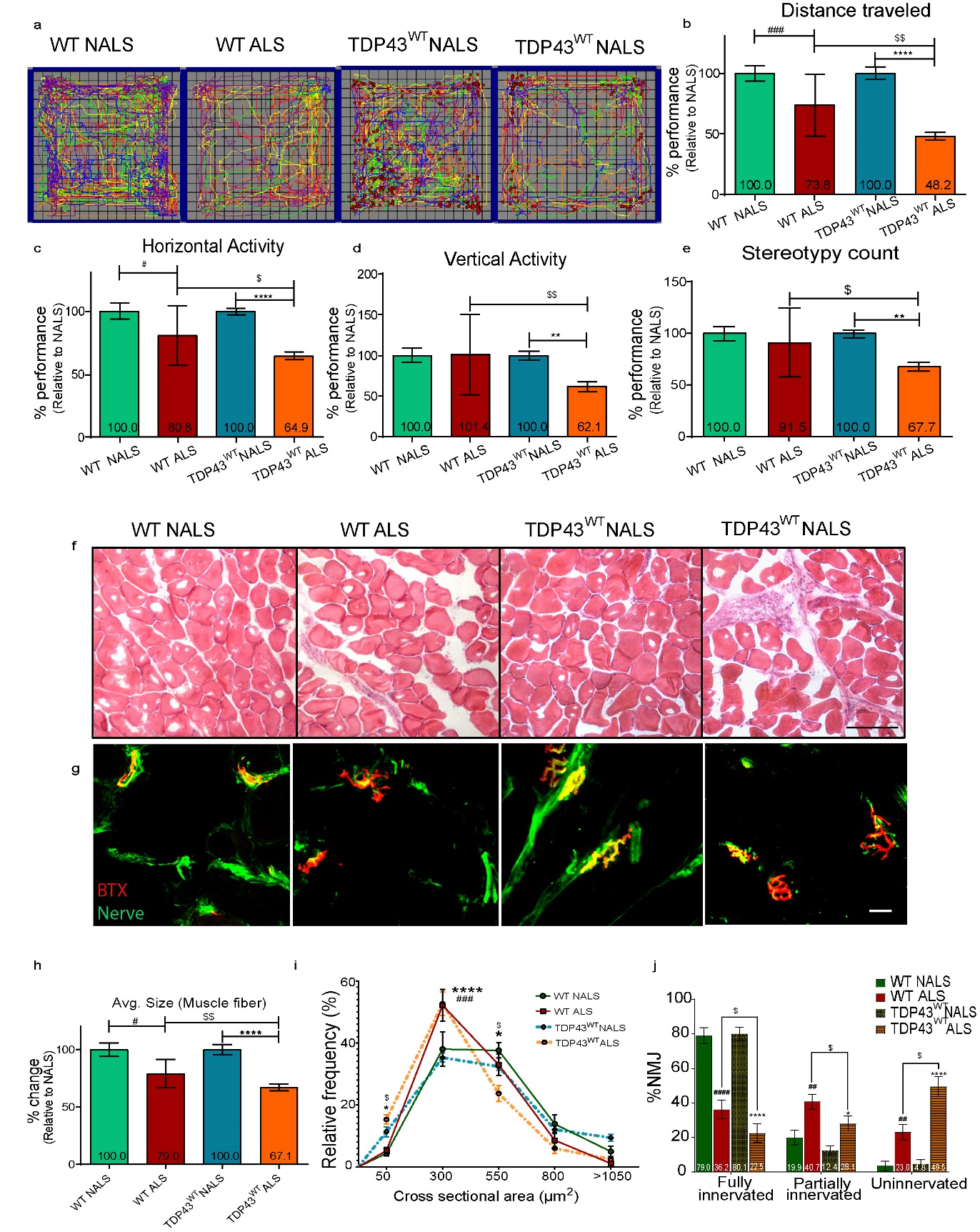


**Supplementary Fig. S2. Normal mice are less affected than hTDP43 transgenic mice to motor dysfunction and muscle damage induced by ALS-CSF infusion.**

(a, e) Effects of ALS-CSF and NALS-CSF on locomotor performance in open field test (OFT) were compared between normal mice and hTDP43 transgenic mice as recipients. (a, b). Graphs c, d and e represent the effects on the horizontal (c) and vertical activity (d), as well as the stereotypy count (e). (n=6). Panel (f) shows changes in the muscle morphology further quantified in the form of average muscle fiber size (h) and the relative frequency of the cross-sectional area (j) across the groups. (Scale bar= 50 µm; n=3). Panel (g) represents the innervation at the NMJs which was quantified and compared across the groups (j). (Scale bar= 20 µm; n=3). Data are mean ± SEM. (#p≤ 0.05 NALS WT v/s ALS WT, *p≤ 0.05 NALS TDP^WT^ v/s ALS TDP^WT^, $p≤ 0.05 ALS WT v/s ALS TDP^WT^ **p≤ 0.01 NALS TDP^WT^ v/s ALS TDP^WT^, $$p≤ 0.01 ALS WT v/s ALS TDP^WT^, ### p ≤ 0.001 NALS WT v/s ALS WT, *** p ≤ 0.001 NALS TDP^WT^ v/s ALS TDP^WT^, and **** p ≤ 0.0001 NALS TDP^WT^ v/s ALS TDP^WT^).


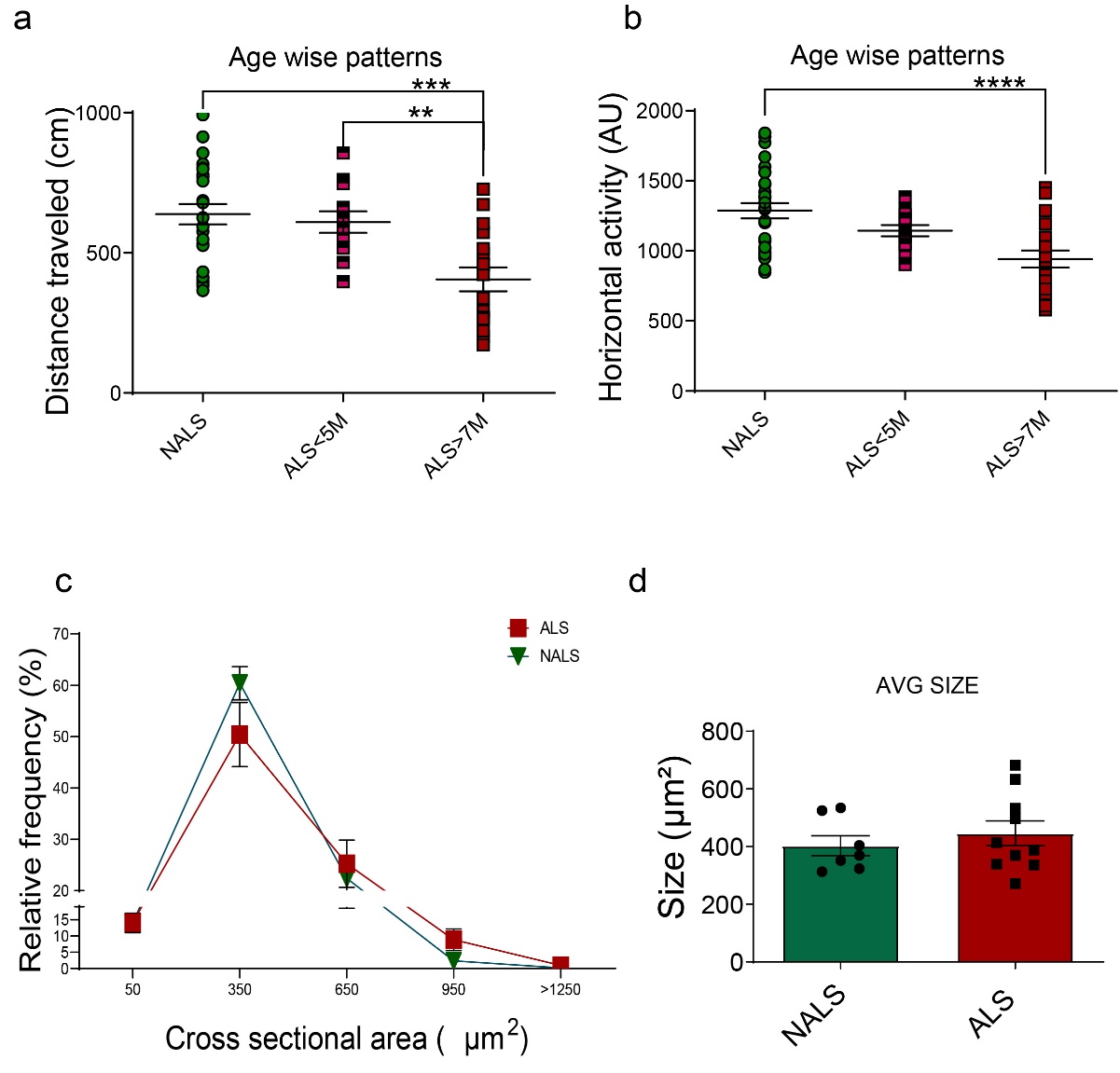
**Supplemental Fig. S2**

**Supplementary Fig. S3. Age-dependent pathology in the hTDP-43 transgenic mice infused with ALS-CSF.**

(a, b) Effects of ALS-CSF on the total distance traveled (a) and horizontal activity (b) on hTDP-43 transgenic mice at 3 to 5 months of age (≤5 M) and 7 to 8 months of age (≥ 7 M)(c,d). No significant changes were observed in the cross-sectional area (c) or the average size (d) of muscle fibres between young TDP43^WT^ mice (less than 5 months of age) administered with NALS-CSF or the ALS-CSF.

**Table S1: List of neuronal peptides with significantly altered translational pattern in response to ALS-CSF**

| **Leading.razor.protein** | **Gene.names** | **NALS/ALS Fold change** | **log2ratio** | **zscore** |
| --- | --- | --- | --- | --- |
| Q8BPW9 | Dnpep | **20.24** | 4.34 | 3.71 |
| A2A815 | Park7 | **5.96** | 2.58 | 2.57 |
| P17751 | Tpi1 | **3.37** | 1.75 | 2.04 |
| P15105 | Glul | **2.02** | 1.01 | 1.56 |
| P16125 | Ldhb | **2.00** | 1.00 | 1.56 |
| P18760 | Cfl1 | **1.83** | 0.87 | 1.47 |
| A0A0N4SVM0 | Capza2 | **1.82** | 0.86 | 1.47 |
| P0DP28 | Calm3 | **1.57** | 0.65 | 1.33 |
| Q04447 | Ckb | **0.77** | -0.38 | 0.66 |
| P99024 | Tubb3;Tubb2b;Tubb2a;Tubb5 | **0.72** | -0.47 | 0.60 |
| Q9CZ13 | Uqcrc1 | **0.67** | -0.58 | 0.53 |
| P63085 | Mapk1 | **0.67** | -0.59 | 0.53 |
| Q9DBJ1 | Pgam1 | **0.64** | -0.64 | 0.50 |
| A0A075B5P4 | Ighg1 | **0.57** | -0.80 | 0.39 |
| F6SVV1 | Gm9493;Rps7 | **0.54** | -0.89 | 0.34 |
| B0R0F0 | Ckmt1 | **0.54** | -0.90 | 0.33 |
| P68372 | Tubb4b;Tubb4a;Tubb3 | **0.53** | -0.91 | 0.32 |
| P07901 | Hsp90aa1;Hsp90ab1 | **0.47** | -1.09 | 0.21 |
| P63260 | Actb;Actg2;Acta1;2;Actg1 | **0.46** | -1.11 | 0.19 |
| D3Z6E4 | Eno2 | **0.46** | -1.14 | 0.18 |
| Q9ERD7 | Tubb3 | **0.46** | -1.14 | 0.18 |
| D3Z6F5 | Atp5a1 | **0.45** | -1.14 | 0.17 |
| Q9DCM0 | Ethe1 | **0.45** | -1.14 | 0.17 |
| P14206 | Rpsa | **0.42** | -1.24 | 0.11 |
| P07724 | Alb | **0.42** | -1.26 | 0.10 |
| P62858 | Rps28 | **0.39** | -1.36 | 0.03 |
| P56480 | Atp5b | **0.38** | -1.39 | 0.01 |
| O08553 | Dpysl2 | **0.34** | -1.55 | -0.09 |
| Q7TMM9 | Tubb2b;Tubb2a | **0.33** | -1.60 | -0.12 |
| P17182 | Eno1 | **0.32** | -1.65 | -0.16 |
| D3Z510 | Aldoa | **0.32** | -1.66 | -0.16 |
| D3Z1Z8 | Stmn1 | **0.31** | -1.68 | -0.17 |
| P63017 | Hspa8;Hspa2 | **0.31** | -1.68 | -0.17 |
| D3YXN6 | Ywhaz | **0.30** | -1.73 | -0.21 |
| D3YWS3 | Pfn2 | **0.28** | -1.82 | -0.26 |
| P50516-2 | Atp6v1a | **0.28** | -1.83 | -0.27 |
| P50518 | Atp6v1e1 | **0.28** | -1.84 | -0.28 |
| Q792Z1 | Try10 | **0.28** | -1.84 | -0.28 |
| Q8BMF4 | Dlat | **0.27** | -1.87 | -0.30 |
| Q9D6F9 | Tubb4a | **0.27** | -1.90 | -0.32 |
| F6RWW8 | Mbp | **0.24** | -2.07 | -0.43 |
| E9PYA3 | Hagh | **0.24** | -2.08 | -0.43 |
| P52480-2 | Pkm | **0.23** | -2.11 | -0.45 |
| P35486 | Pdha1 | **0.22** | -2.18 | -0.50 |
| Q99KI0 | Aco2 | **0.18** | -2.45 | -0.68 |
| P20357 | Map2 | **0.16** | -2.62 | -0.78 |
| P19157 | Gstp1 | **0.16** | -2.62 | -0.79 |
| P17742 | Ppia;Gm5160 | **0.16** | -2.68 | -0.82 |
| A2AQR0 | Gpd2 | **0.14** | -2.79 | -0.89 |
| A0A0G2JGX4 | Atp1a3;Atp1a2;Atp1a1 | **0.14** | -2.80 | -0.90 |
| Q01853 | Vcp | **0.13** | -2.92 | -0.97 |
| A0A1D5RLD8 | Gapdh;Gm3839 | **0.13** | -2.92 | -0.98 |
| B7FAU8 | Gdi1 | **0.13** | -2.95 | -0.99 |
| Q60932-2 | Vdac1 | **0.12** | -3.06 | -1.07 |
| P68254-2 | Ywhaq | **0.11** | -3.19 | -1.15 |
| D3Z6I8 | Tpm3;Tpm3-rs7 | **0.11** | -3.25 | -1.19 |
| P60867 | Rps20 | **0.10** | -3.30 | -1.23 |
| P08249 | Mdh2 | **0.10** | -3.33 | -1.24 |
| O08599 | Stxbp1 | **0.10** | -3.37 | -1.27 |
| A2A5N1 | Ywhab | **0.09** | -3.46 | -1.33 |
| P60761 | Nrgn | **0.09** | -3.52 | -1.37 |
| P14152 | Mdh1 | **0.09** | -3.54 | -1.38 |
| P61982 | Ywhab;Ywhag | **0.09** | -3.55 | -1.39 |
| A2ALV1 | Sh3gl2 | **0.06** | -4.08 | -1.73 |
